# Behavior of Partially Grouted Concrete Masonry Walls under Quasi-Static Cyclic Lateral Loading

**DOI:** 10.3390/ma13102424

**Published:** 2020-05-25

**Authors:** Sebastián Calderón, Laura Vargas, Cristián Sandoval, Gerardo Araya-Letelier

**Affiliations:** 1 Department of Structural and Geotechnical Engineering, Pontificia Universidad Católica de Chile, Vicuña Mackenna 4860, Macul, Santiago 7820436, Chile; sacalder@uc.cl (S.C.); lavargas3@uc.cl (L.V.); 2 Department of Architecture, Built Environment and Construction Engineering, Politecnico di Milano, Piazza Leonardo da Vinci 32, 20133 Milan, Italy; 3 School of Architecture, Pontificia Universidad Católica de Chile, El Comendador 1916, Providencia, Santiago 7520245, Chile; 4 School of Civil Construction, Pontificia Universidad Católica de Chile, Vicuña Mackenna 4860, Macul, Santiago 7820436, Chile; gerardo.araya@uc.cl

**Keywords:** partially grouted masonry, concrete block, in-plane cyclic testing, shear response, bed-joint reinforcement

## Abstract

Eight partially grouted (PG-RM) concrete masonry walls were tested to study the influence of the strength and width of blocks, the wall aspect ratio, the horizontal and vertical reinforcement ratio, and the presence of edge elements (flanges). The results were analyzed in terms of the failure mode, damage progression, shear strength, lateral stiffness degradation, equivalent viscous damping ratio, and displacement ductility. Additionally, the performances of some existing shear expressions were analyzed by comparing the measured and predicted lateral load capacity of the tested walls. Based on the results, a slight increment in the lateral stiffness was achieved when employing stronger blocks, while the shear strength remained constant. Besides, increasing the width of concrete blocks did not have a significant effect on the shear strength nor in the initial tangential stiffness, but it generated a softer post-peak strength degradation. Increasing the wall aspect ratio reduced the brittleness of the response and the shear strength. Reducing the amount of vertical reinforcement lowered the resulting shear strength, although it also slowed down the post-peak resistance degradation. Transversal edge elements provided integrity to the wall response, generated softer resistance degradation, and improved the symmetry of the response, but they did not raise the lateral resistance.

## 1. Introduction

Hollow concrete block (HCB) partially grouted reinforced masonry (PG-RM) is an earthquake-resistant system that is common in several countries. This masonry typology, together with confined masonry, has shown good seismic performance in recent seismic events [1]. It should be noted that, as PG-RM shear walls consist of unreinforced masonry panels within grouted cores, they can be compared with confined masonry to some extent. However, the similarities between the construction systems depend on the spacing of the vertical grouted cores that surround the panels. Less spaced cores, as in partially grouted masonry, provide redundancy and more uniform distributions of lateral forces [2].

The seismic behavior of PG-RM walls has received relatively limited research despite its prevalence in the construction market. Much of the most recent research effort has been devoted to understanding the role of different design variables on the seismic behavior of PG-RM walls, as well as proposing shear strength equations for design purposes. In this context, several authors (e.g., [3,4,5,6,7,8,9]) have developed different expressions and methodologies for estimating the shear resistance of HCB PG-RM shear walls. However, most of those studies have focused on shear walls with bond-beam reinforcement. Employing such reinforcement type implies the use of U-shaped blocks, whose internal configuration provides space that is used for placing cement grout and steel reinforcing bars. PG-RM walls with bond-beam reinforcement (BBR) are common in North America and New Zealand, and its behavior is different in comparison to PG-RM walls with bed-joint reinforcement [10,11,12]. Using bed-joint reinforcement is a more common construction practice in Central and South America, which means that the steel bars are embedded in horizontal mortar joints.

As mentioned, the available experimental evidence on PG-RM shear walls made of HCB and with bed-joint reinforcement (BJR) is more limited and less heterogeneous in comparison with the available for PG-RM shear walls with BBR [6,7]. This fact illustrates the need to enlarge the experimental database on BJR-PG-RM shear walls built with HCB. In fact, according to the best of the authors’ knowledge, a total of 182 specimens of BJR PG-RM shear walls of HCB have been tested to date [13,14,15,16,17,18,19,20,21,22,23,24,25,26,27], most of them in North America (51.6%), followed by Europe (28.6%), and Central and South America (19.8%). Those studies have mainly focused on determining the influence of the aspect (height-to-length) ratio, horizontal and vertical reinforcement ratio, and axial load.

Overall, it has been reported that incrementing the wall aspect ratio can change the failure mode of walls [26]. However, contradictory effects have been reported on the influence of the wall aspect ratio in the shear strength. For instance, Lüders and Hidalgo [16] and Ramírez et al. [21] observed that incrementing the wall aspect ratio leads to a decrement on the shear strength, in contrast to the findings of Schultz et al. [22]. In addition, Ramírez et al. [21] indicated that the wall aspect ratio and the deformation capacity are proportional, but Schultz et al. [22] observed the opposite. On the other hand, Ramírez et al. [21] and Schultz et al. [22] coincided in that the wall aspect ratio and the energy dissipation capacity are inversely correlated. In any case, these contradictory results could have been influenced by how the cross-section area is considered in the shear stress calculations, as already commented by Sandoval et al. [28]. For example, some experimental works on PG-RM walls reported the shear stress over the total cross-section area (gross area) and others over the net-cross section area (total area discounting the ungrouted hollows).

Regarding axial load, this variable has a positive effect on the shear strength [14,19,21,25], although higher axial loads are associated with more brittle behaviors and lower deformation capacities [18,25]. In addition, Ramírez et al. [21] indicated that the energy dissipation capacity increases with the axial load. Differently, the effect of axial loads on the crack patterns is not clear since Oan [19] observed variations in the crack-widths and distribution, whereas Meli et al. [17] and Ramírez et al. [21] only observed reductions on the crack-widths.

The effect of the horizontal reinforcement ratio has been the most studied design parameter. In general, it has been reported that the presence of BJR is beneficial for the in-plane behavior of PG-RM shear walls because it provides post-cracking integrity [22,26], as well as capability of energy dissipation and ductility [14,24]. Tomaževič and Lutman [26] indicated that horizontal reinforcement acts only when the panel is diagonally cracked and that its effectiveness depends on the capacity of materials to provided appropriated local anchoring to reinforcement. The authors also pointed out that an increment in the horizontal reinforcement ratio from 0.14% to 0.50% in slender walls (height-to-length ratio of 2.30) shifted the failure mode from shear to flexural. On the other hand, the effect of the horizontal reinforcement on the shear strength is still a subject of research because some authors have observed that shear strength increments with a higher horizontal reinforcement ratio [14,16,21], while others have stated that there is no relationship between them [19,27]. In this aspect, the main function of evenly distributed reinforcement (BJR) is to provide connectivity among cracked zones that usually concentrate in unreinforced sectors of panels [17,19] and ungrouted zones nearby grouted cores [19].

It has been reported that BJR can be used to provide the same shear strength and ductility than with bond-beam reinforcement [13], and that walls with BJR exhibited more distributed and thicker cracks than bond-beam reinforced walls [13,19,24]. Nonetheless, the main drawback of BJR is that the diameter of reinforcement bars is limited by the joint thickness since the use of an excessive diameter could generate a weak horizontal plane and reduce the shear strength [19]. On the other hand, the majority of tests considered a high vertical reinforcement ratio ($\rho_{v}$) in an attempt to avoid a flexural failure mode (e.g., $\rho_{v}>0.4\%$), although walls with vertical reinforcement ratios closer to design values (e.g., $\rho_{v}\approx 0.05\%$) could experience lower resistances [23].

In general, the studies mentioned above have reported the individual influence of design parameters, that is to say, analyzed specimens had variations in one variable at the time. However, some authors have also reported combined effects of more than one design parameter. Tomaževič et al. [29] reported that the effectiveness of the horizontal reinforcement (measured as a percentage of the yield strength) decreases with a higher axial load. On the other hand, Schultz et al. [22] indicated that the effect of the horizontal reinforcement on the shear strength depends on the wall aspect ratio. In addition, Ramírez et al. [21] pointed out that the effect of the axial load on the shear strength is higher with decreasing levels of wall aspect ratios. Nonetheless, there is limited information about combined effects, and, therefore, more experimental programs are required for their characterization.

Other variables, such as loading protocol or construction methods, have also been studied, but have received scarce attention. Tomaževič et al. [29] reported that a statically-applied alternated lateral loading protocol is suitable to avoid overestimating the lateral resistance of specimens, and to measure their seismic response parameters, such as the energy dissipation capacity. Oan [19] concluded that walls in which the joint reinforcement was laid on the dry joint and then covered with mortar exhibited the same behavior as walls in which reinforcement was placed between two layers of joint mortar.

Although several experimental programs have been carried out to date, the influence of different design variables on the shear behavior of BJR PG-RM shear walls made of HCB continues being a subject of research. Given the above, the main novelty of this paper resides in experimentally investigating some of the design variables that have not been studied yet. Thus, this research presents the experimental results of eight tests of full-scale BJR PG-RM shear walls made of hollow concrete blocks, subjected to constant axial load and alternated in-plane lateral loads. The experimental program analyzed the influence of the compressive strength and width of the HCB, the wall aspect ratio, the horizontal and vertical reinforcement ratio, and the presence of flanges (edge transversal elements). The comparison was performed by considering a base design, over which one experimental variable was modified per test. In addition, two base walls were tested to observe the variability of the response. The results were analyzed in terms of damage progression, hysteresis curves, and seismic performance indicators (degradation of lateral stiffness, equivalent viscous damping ratio, and ductility). Additionally, the accuracy of some selected expressions was evaluated by estimating the resistance of the tested walls.

## 2. Experimental Program

### 2.1. Description of Specimens

**Figure 1:**
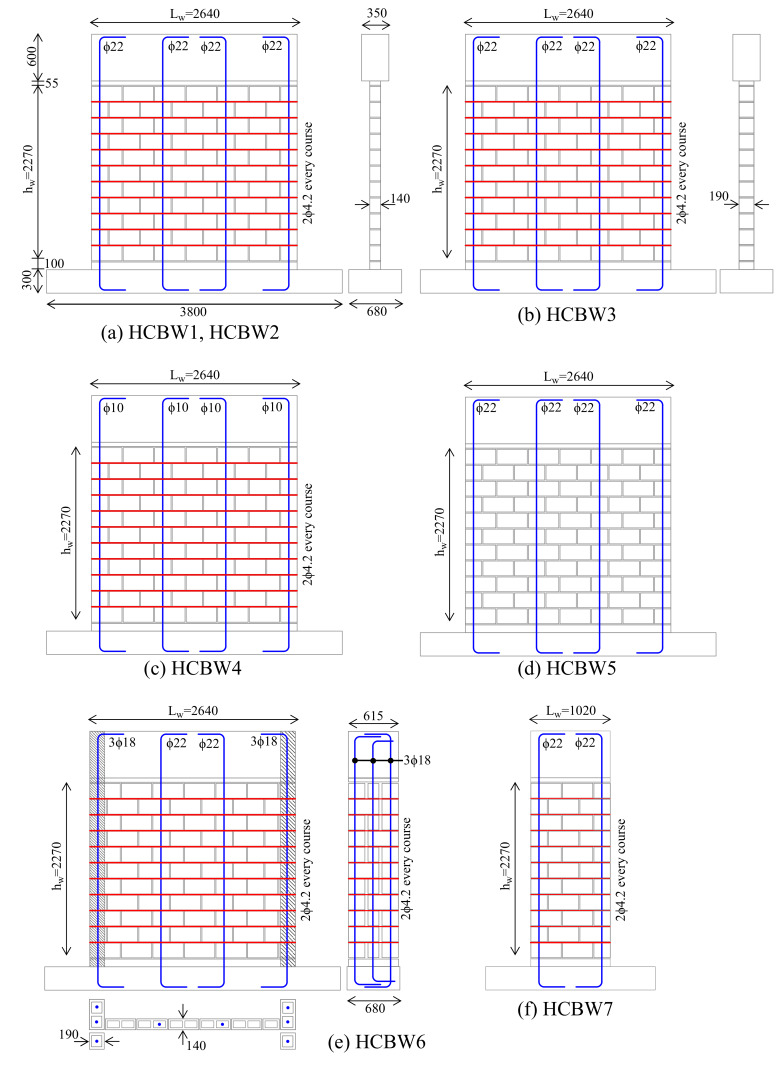
Tested BJ-PG-RM wall schemas: (**a**) walls HCBW1 and HCBW2, (**b**) wall HCBW3, (**c**) wall HCBW4, (**d**) wall HCBW5, (**e**) wall HCBW6, and (**f**) wall HCBW7. Dimensions in mm.

The experimental program consisted of eight PG-RM shear walls built with HCB, two of them were employed as base-design and the remaining six walls implemented one variation in their design parameters. The base-design was replicated in two walls to observe possible variations in their response. The design variables under study were the strength and width of blocks, the wall aspect ratio, the vertical and horizontal reinforcement ratio, and the presence of flanges (transversal edge elements). Table 1 summarizes the relevant properties of walls and Figure 1 schematizes their design. It is worth noting that the block compressive strength ($f_{cu}$) and masonry compressive strength ($f_{m}^{\prime }$) were calculated from experimental data over the gross cross-section of concrete blocks, as detailed in Section 2.3. Axial pre-compression stress ($\sigma_{n}$) was calculated as a function of the masonry compressive strength ($f_{m}^{\prime }$). The horizontal reinforcement ratio ($\rho_{h}$) was calculated as the total area of horizontally oriented reinforcement divided by the vertical gross section area (height by block width) and the vertical reinforcement ratio ($\rho_{v}$) as the total area of vertically oriented reinforcement divided by the gross cross-section area of each wall.

**Figure 2:**
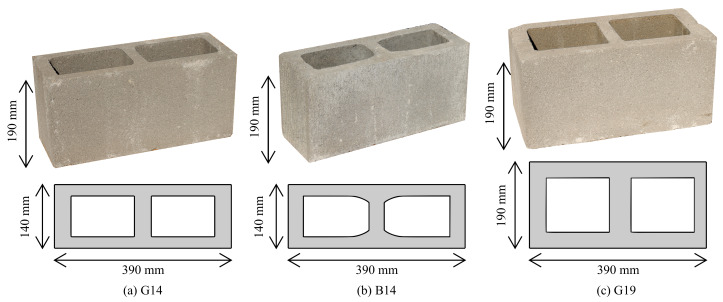
Employed hollow concrete block types: (**a**) G14, (**b**) B14, and (**c**) G19.

Walls HCBW1-A and HCBW1-B represent the base-design (Figure 1a). HCBs of 390 mm in length, 190 mm in height, and 140 mm in thickness (Figure 2a) were employed to build masonry wall panels that were 2270 mm tall. Most of the wall panels were horizontally reinforced in every mortar bed-joint with ladder-type reinforcement made of 2 longitudinal steel bars of 4.2 mm in diameter and vertically reinforced with four steel rebars of 22 mm in diameter. The reinforcement provided to the wall panels a vertical ($\rho_{v}$) and horizontal reinforcement ratio ($\rho_{h}$) equal to 0.41% and 0.087% (based on gross areas), respectively. It is important to remark that a high amount of vertical reinforcement was provided to induce a shear (diagonal tension) failure mode. In addition, reinforced concrete footings of 680 mm in width and 300 mm in height were built to fix the specimens to the laboratory strong floor and to ensure an appropriate anchoring to vertical reinforcement. Capping beams 350 mm thick and 600 mm tall were cast to impose the displacement at the top of specimens uniformly and to ensure an appropriate anchoring to vertical reinforcement bars. These design specifications remained constant in the other walls with the exception of the design variable under study in each specimen.

Wall HCBW2 (Figure 1a) was built with the B14-HCB depicted in Figure 2b, which had a higher compression and tension strength than the block employed in the base walls. Although the internal geometry of B14 and G14 blocks are slightly different, the external dimensions were the same. More details on the material properties of units are provided in the following sections.

Wall HCBW3 (Figure 1b) was wider than the base walls since HCBs of 190 mm in width (Figure 2c) were employed in its construction. The vertical reinforcement ratio of Wall HCBW3 ($\rho_{v}$) was reduced by about 26.3% compared to the base wall because of the variation in its cross-section, but this ratio was still high enough to force the diagonal tension failure mode. The horizontal reinforcement ratio ($\rho_{h}$) was also 26.3% lower than in the base wall. Both effects are discussed in the following sections.

Wall HCBW4 (Figure 1c) had four steel bars of 10 mm in diameter as vertical reinforcement, although the same arrangement of the base-walls was kept. Vertical reinforcement ratio ($\rho_{v}$) was 0.08%, which represents a reduction of 80.5% with regards to the base-cases, but still, this ratio was slightly above the minimum of 0.05% prescribed by Chilean design provisions [30].

Wall HCBW5 did not have horizontal reinforcement, as shown in Figure 1d. Although this wall was not a reinforced masonry wall strictly, it was used to study the contribution of vertical reinforcement and grouted cores to the lateral resistance.

The HCBW6 (Figure 1e) had transversal elements in both edges, which provided an I-shaped cross-section to the wall. Edge elements were 190 mm thick and 615 mm long and were materialized with the same HCBs of 190 mm in width (Figure 2c) that were employed in Wall HCBW3. Notice that the in-plane masonry panel was appropriately connected with edge elements with a running bond and with the horizontal reinforcement that was extended into the edge elements. In this regard, it was considered that the horizontal reinforcement ratio ($\rho_{h}$) in the middle of the panel was representative of the horizontal reinforcement ratio of the wall. In addition, vertical reinforcement bars at edges of the panels were three bars of 18 mm in diameter in order to preserve the vertical reinforcement ratio ($\rho_{v}$) calculated over gross cross-section with respect to the base-case design. Moreover, some modifications were done to the footing and capping beams in order to provide appropriate anchoring to vertical reinforcement bars.

The HCBW7 wall (Figure 1f) was 1020 mm in length, with a wall aspect ratio of 2.23. One vertical steel bar 22 mm in diameter was placed at each edge of the wall to keep the same reinforcement ratios as the base wall.

### 2.2. Experimental Setup

**Figure 3:**
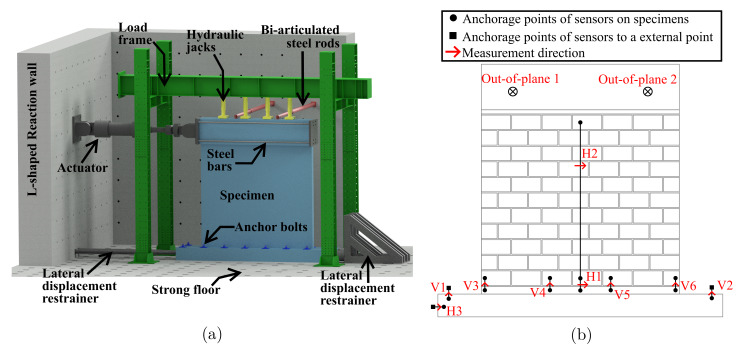
Testing setup: (**a**) general view; and (**b**) instrumentation.

Alternated in-plane lateral displacements and constant axial load were imposed on the constructed specimens, under a cantilever boundary condition. The experimental setup is depicted in Figure 3a. The footing of each specimen was fixed to the strong floor of the laboratory with high-strength steel anchor bolts of 36 mm in diameter every 500 mm. The bolts were placed at the front and back faces of the specimens, as depicted in Figure 3a. In addition, lateral restrainers were used to limit any rigid body sliding movement. Besides, possible out-of-plane displacements were restricted at the mid-height of the capping beam with two bi-articulated steel rods that reacted against the rear section of the L-shaped reaction wall.

Lateral displacements were applied by a servo-controlled hydraulic actuator with capacities of 1000 and 800 kN in the push and pull load directions, respectively. One edge of the actuator was connected to the reinforced concrete beam in the top of the wall using four pre-stressed high-strength steel rods of 36 mm diameter and steel plates of 50 mm in thickness. The other edge of the actuator reacted against the L-shaped reinforced concrete reaction wall of the laboratory. Axial load was applied by means of four hydraulic jacks of 25 kN of capacity. All of them were evenly spaced and connected to the same oil pump in order to apply uniform axial stress to masonry panels. One side of those jacks was fixed to the steel reaction frame, as depicted in Figure 3a, and the other was attached to steel roller packs that allowed transferring the axial load without introducing overturning moments to jacks.

Elevent linear variable differential transducers (LVDTs) were employed to register movements at different locations of the specimens. These locations on walls are presented in Figure 3b. Sensors V1 and V2 were employed to measure the vertical movements of the bottom head beam and, similarly, sensors V3–V6 were used to monitor the unstick of the first mortar joint with respect to the reinforced concrete of the foundation. Out-of-plane displacements were measured with two LVDTs, at the same locations the bi-articulated steel rods were connected to capping beams. Regarding horizontal displacements, sensor H1 measured the relative displacements between the foundation and first course, and sensor H2 between the first course and last courses. Additionally, LVDT H3 recorded the rigid body sliding of the specimen. On the other hand, strain gauges were also used in Wall HCBW1-A, but more details are provided in the following sections.

**Figure 4:**
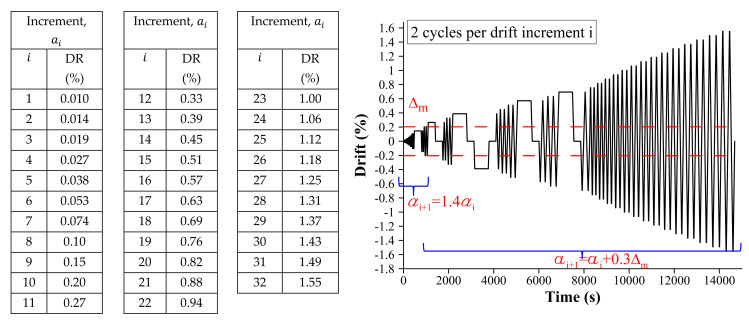
Drift protocol employed in tests.

The same protocol of imposed lateral displacement was employed for all walls, which was defined in accordance with FEMA 461 [31]. The protocol is a function of the drift ratio (DR) (ratio between horizontal deformation and height of the wall), and its displacement history is shown in Figure 4. The semi-amplitude ($a_{i+1}$) for a drift increment i+1 was calculated with Equation (1), considering the parameters $\Delta_{0}$, $\Delta_{m}$, and *n* as 0.01%, 0.24%, and 10, respectively. As can be observed, two complete cycles per drift increment were performed to study the degradation of the response under equal repetitive displacements. Moreover, the protocol considered some pauses to map cracks and damage progression. In addition, displacements were applied quasi-statically with a speed of 0.25 mm/s. It is important to mention that cyclic quasi-static protocols are appropriate to characterize maximum resistance and estimate seismic properties [29].

(1) $$\begin{array}{c} a_{i+1}=\left\{\begin{array}{cc} 1.4\cdot a_{i} & \mathrm{if}\;a_{i+1}\leq \Delta_{m}\\ a_{i}+0.3\cdot \Delta_{m} & \mathrm{if}\;a_{i+1}>\Delta_{m} \end{array}\right.\;,a_{i}=\Delta_{0} \end{array}$$

### 2.3. Material Properties

As previously mentioned, three types of HCB were employed in the construction of specimens (Figure 2), whose geometrical and mechanical properties (i.e., compression ($f_{cu}$) and tensile ($f_{tu}$) strength and Young’s modulus ($E_{u}$)) are presented in Table 2. These properties were experimentally measured in accordance with Chilean provisions [32]. It is worth noting that all values were calculated considering the gross area of blocks. Additionally, all employed blocks were manufactured with low-fluidity extruded concrete. HCBs G14, and G19 (Figure 2a,c) were manufactured by the same company, and, therefore, they had a similar internal shape, voids ratio, and were made of a similar concrete mixture. Although HCB G19 presented an average compressive strength 21.6% higher than the HCB G14, the observed difference was not significant because of the coefficient of variation (CV) of the results. Moreover, HCBs G14 and G19 had almost similar average tensile strength. These results confirm that these HCBs were made with a similar concrete mixture, as indicated by the manufacturer. On the other hand, HCB B14 (Figure 2b) had a lower voids ratio ($\nu_{r}$) than the other block types and exhibited the highest compressive and tensile strength. Notice that HCB G19 had the highest Young’s modulus, which can be a consequence of their lower height-to-width ratio that might provide a higher degree of confinement during compressive tests.

The same mortar and grout were employed in all walls, whose compressive properties were measured on RILEM type specimens (160 mm × 40 mm × 40 mm) following Chilean provisions [33]. Mortar and grout presented compressive strengths of 22.0 (CV = 13.4%) and 61.8 MPa (CV = 12.5%) and Young’s moduli of 14.7 (CV = 14.2%) and 23.9 GPa (CV = 7.6%), respectively. The latter values satisfied the recommendation that grout should be strong enough to appropriately connect and transfer stresses between vertical reinforcement bars and blocks.

Three blocks stack bond-prism compression tests were performed in accordance with Chilean masonry design code NCh1928 [30], considering a minimum of three prisms per block type. From them, masonry compressive strength ($f_{m}^{\prime }$) and Young’s modulus ($E_{m}$) were measured. The results are presented in Table 3, where it can be seen that the strongest and the stiffest masonry composite was made of the strongest HCB (B14). On the other hand, prisms of blocks of G14 were slightly stronger than prisms of blocks of G19, despite blocks of G19 exhibiting higher compressive strength than blocks of G14. This result might corroborate the possible confinement effect observed on the compression tests of blocks. However, the masonry made of blocks of G19 presented a higher Young’s modulus than the masonry of blocks of G14.

**Figure 5:**
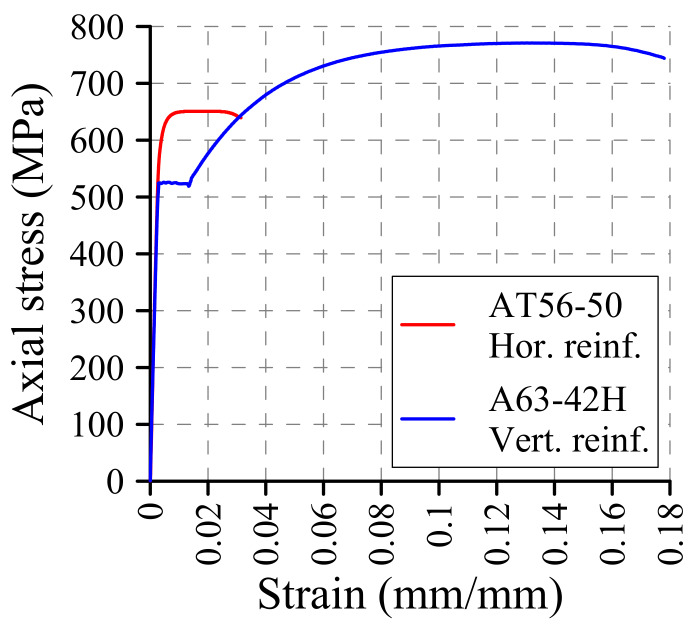
Typical axial response of steel reinforcement.

Vertical reinforcement bars were hot-rolled steel bars of quality A63-42H, while horizontal reinforcement elements were made of cold-drawn steel wire of quality AT56-50. Different steel qualities were employed according to Chilean local construction practices. Specimens of both steel types were characterized by means of tensile tests, which were carried out according to Chilean provisions [34]. AT56-50 and A63-42H had Young’s modulis of 206.0 (CV = 5.1%) and 196.4 GPa (CV = 2.8%), yield strains of 3082 (CV = 1.2%) and 2700 μm/m (CV = 5.4%), yield strengths of 644.9 (CV = 5.0%) and 521.3 MPa (CV = 0.7%), and tensile strengths of 667.0 (CV = 2.7%) and 769.2 MPa (CV = 0.4%), respectively. Representative stress–strain curves are presented in Figure 5. As can be observed, steel AT56-50 had a higher yield strength than A63-42H, but it is less ductile and did not present a yield plateau or hardening.

## 3. Test Results

### 3.1. Hysteric Response

**Figure 6:**
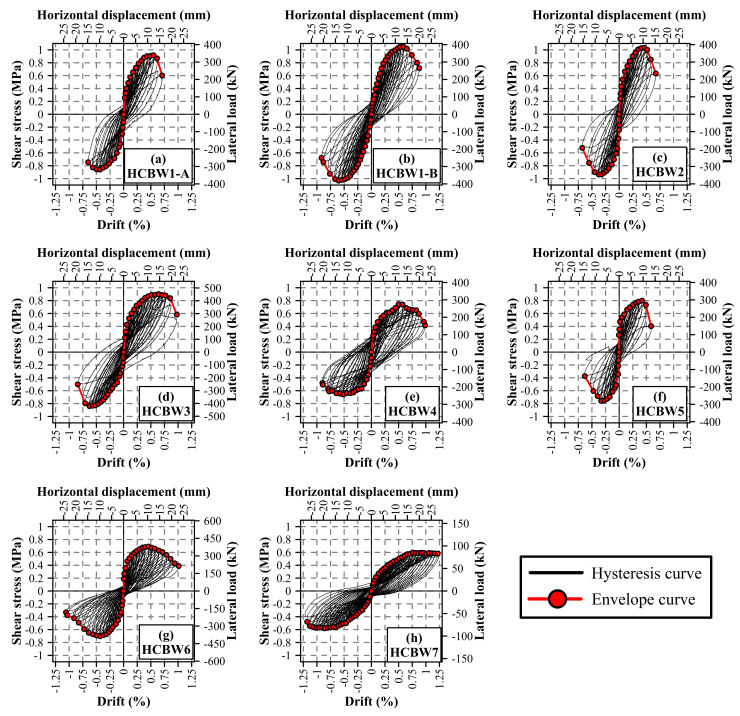
Hysteresis curves of tested walls: (**a**) wall HCBW1-A, (**b**) wall HCBW1-B, (**c**) wall HCBW2, (**d**) wall HCBW3, (**e**) wall HCBW4, (**f**) wall HCBW5, (**g**) wall HCBW6, and (**h**) wall HCBW7.

The in-plane hysteresis curves registered during the tests are presented in Figure 6, and the information (lateral displacement, drift, lateral force, and shear stress) regarding the occurrence of the first major diagonal crack ($\delta_{1cr}$, $\Delta_{1cr}$, $V_{1cr}$, and $\tau_{1cr}$, respectively) and the maximum lateral load ($\delta_{Vmax}$, $\Delta_{Vmax}$, $V_{max}$, and $\tau_{g,max}$, respectively) for both loading directions is reported in Table 4. In general, all walls had hysteresis curves with similar shapes in both loading directions, although maximum loads were measured in the push loading direction in all wall specimens. In addition, an initial elastic stage can be distinguished in all cases, in which hysteresis cycles were narrow, and specimens remained almost undamaged. This elastic stage was limited to a few drifts increments, which corresponded to values between 0.8% and 8.2% of the drift at maximum resistance.

Afterward, progressive degradation of the lateral stiffness can be identified in all walls, where hysteresis cycles became wider as the deformation incremented. In this stage, the first major diagonal crack was identified on the specimens, associated with deformations that ranged from 1.25 to 6.58 mm. These values represented a fraction between 12.4% and 56.3% of the deformation of the maximum load of the corresponding load direction. However, it is important to remark that the major crack could only be observed once it achieved a considerable width. Therefore, this observation does not strictly correspond to the end of the elastic behavior but sets an upper bound and provides information when a certain degree of damage has been reached.

It can be noticed that the highest cracking resistance in each specimen was not always observed in the push loading direction, unlike the maximum resistance. Moreover, the lowest cracking deformation and cracking resistance of each specimen were not always in the same loading direction in all specimens. Therefore, the loading directions of smaller cracking deformation, maximum cracking resistance, and maximum shear strength are not correlated. Besides, it does not seem that the identification of the first major diagonal crack in one loading direction triggers the diagonal cracking in the opposite loading direction because of the ratios between maximum and minimum cracking deformations ($\delta_{1cr}^{max}/\delta_{1cr}^{min}$) ranged from 104.3% to 200.6%.

It can be considered that the progression of damage was relatively uniform up to the maximum lateral resistance was reached. Then, a strong decay in the lateral resistance was observed in most specimens, with the exception of Walls HCBW6 and HCBW7. In Wall HCBW6, the softer degradation was produced by the edge elements that formed a frame that bounded the interior masonry panel. Wall HCBW7 experienced a gradual degradation because of its higher wall aspect ratio.

As mentioned, maximum resistances were achieved in the push loading direction, with values that ranged from 0.69 to 1.05 MPa and were from 0.0% to 10.1% higher than in the pull loading direction. It is also important to mention that the loading direction in which the maximum load was achieved was not always the same that exhibited the largest associated lateral deformation. Moreover, reaching the maximum load in one loading direction apparently produced the same phenomena in the opposite loading direction, due to the ratios between deformations at maximum resistances in both loading directions ($|\delta_{Vmax}^{-}|/\delta_{Vmax}^{+}$) ranged between 101.3% and 127.7%. This interval is smaller than the observed for the first major diagonal crack, where a contrary effect was noticed.

**Figure 7:**
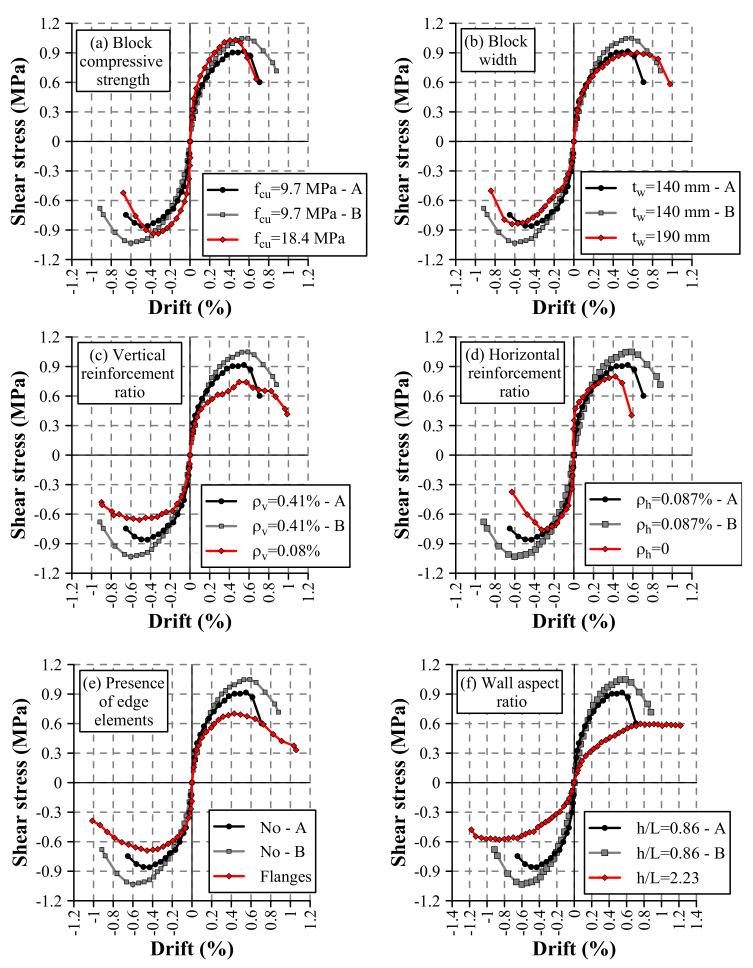
Comparison of envelope curves of walls with different: (**a**) block compressive strength, (**b**) block width, (**c**) vertical reinforcement ratio, (**d**) horizontal reinforcement ratio, (**e**) presence of edge elements, and (**f**) wall aspect ratio.

Envelope curves were calculated by connecting the points of the hysteresis curves that exhibited the maximum force in the push loading direction and the minimum force in the pull loading direction for the first cycle of each displacement increment. Figure 7 superimposes the obtained curves in order to compare the effects of the different studied design variables. It is important to mention that Walls HCBW1-A and HCBW1-B are depicted in all subplots in black and gray lines, respectively, while variations with regard to the base-design are plotted in red lines. As appreciated, Walls HCBW1-A and HCBW1-B had a similar initial response. However, Wall HCBW1-A exhibited a weaker response than Wall HCBW1-B, with maximum lateral resistance and lateral displacement 12.6% and 6.7% lower, respectively. Besides, similar differences can be distinguished in the pull loading direction. Therefore, even though the same design properties, material qualities, construction procedures, and testing conditions were used, the intrinsic variability of the material may cause notorious variations in the response results, as also indicated by Oan [19] and Hoque [15].

Figure 7a shows the walls with different block compressive strength. The figure indicates that Wall HCBW2 had a stiffer response, which resulted in a maximum resistance at lower deformation than Walls HCBW1-A and B, in both loading directions. Nonetheless, non-significant differences can be observed in the shear strength nor post-peak behavior. The stiffer response of Wall HCBW2 can be explained since prisms made of blocks of B14, the ones employed in this wall, had a higher average Young’s modulus than the prisms made of blocks of G14, the ones used in the base walls. On the other hand, it is known that diagonal tension (shear) failure mode occurs when cracks propagate along with the weaker zones among blocks and block–mortar interfaces. In this particular case, base blocks (G14) were stronger enough to force cracks ran along with block–mortar interfaces. Nonetheless, employing a stronger block does not necessarily improve the bond strength between mortar and blocks. Therefore, improving blocks quality does not necessarily correspond with an enhanced in-plane behavior, a situation that might explain the insensitiveness of the wall response to the strength of the block (in compression and tension).

The effect of the block width is presented in Figure 7b, where there were no significant variations on the shear strength nor in the initial stiffness. It is important to notice that, even though shear strength of Walls HCBW1-A, HCBW1-B, and HCBW3 are similar, the maximum lateral force of Wall HCBW3 was higher than the corresponding to the reference walls due to its higher gross cross-section. Unlike Walls HCBW1 A and B, Wall HCBW3 exhibited a softer degradation of stiffness since achieving the elastic limit in the push direction. Indeed, this wall held 90% of its maximum shear strength between drifts of 0.34% and 0.77%, equivalent to displacements of between 7.7 and 17.5 mm. Nonetheless, this effect only happened in the push loading direction, which is attributed to the pre-damage generated by imposed displacements on the contrary loading direction. Despite the softer degradation in the push direction, Walls HCBW1-A, HCBW1-B, and HCBW3 reached their maximum resistance at comparable deformations. Therefore, it seems that employing a thicker block allowed to redistribute tensions once damage occurs, which provides integrity to the panel. However, this effect was limited due to the brittleness of the blocks.

On the other hand, significant effects can be attributed to the variations of the vertical and horizontal reinforcement ratios, the wall aspect ratio, and the presence of flanges. As can be observed in Figure 7c, decreasing the amount of vertical reinforcement had a patent impact on the elastic limit and the shear strength, but no in the deformation corresponding to the maximum resistance. In this regard, the shear strength of Wall HCBW4 in the push direction was 24.3% lower than the average of reference walls. Despite this, the walls exhibited similar stiffness for drifts lower than 0.1%. Afterward, when vertical reinforcement bars started to work, Wall HCBW4 developed lower forces than the reference walls for the same deformation, which was a consequence of the lower vertical reinforcement section that provided a lower flexural stiffness to Wall HCBW4. The reduced vertical reinforcement also impacted the post-peak behavior. Wall HCBW4 exhibited a decrement of 20% in its maximum lateral resistance at a drift 73.4% higher than the drift at maximum resistance ($\Delta_{Vmax}$). Nonetheless, Walls HCBW1-A and HCBW1-B showed a reduction of 20% in their lateral force at drifts 20.6% and 37.6% higher than the corresponding drifts at maximum resistance ($\Delta_{Vmax}$), respectively. This better post-peak response of Wall HCBW4 can be explained by the fact that this wall had a higher contribution of the flexural mode than the reference walls, and a flexural failure mode is less brittle than a shear-dominated failure. Additionally, a higher section of vertical rebars increments the axial stresses transferred to the masonry composite by the tensioned vertical bars, and it has reported a brittler failure mode when axial load is higher [21]. On the other hand, the lower shear strength observed on Wall HCBW4 was possibly due to the reduction of the contribution of the dowel effect of vertical steel rebars and a lower indirect axial pre-compression (the axial stress induced by the elongation of the vertical rebars).

Figure 7d shows the effect of the horizontal reinforcement ratio, where Wall HCBW5 achieved a lower lateral resistance at a lower deformation than the reference walls. Moreover, Wall HCBW5 exhibited a sudden drop in its post-peak lateral resistance. This situation confirms the findings regarding the ability of ladder type-reinforcement to increase the shear strength [14,16,21] and to provide integrity to the compound once cracks have appeared [22,26].

Figure 7e compares the reference walls with Wall HCBW6, which had edge elements. As can be observed, the wall with flanges achieved a shear strength 29.3% lower than the average between reference walls. Nonetheless, maximum lateral forces were very similar, since the HCBW6 resistance was between the corresponding values of Walls HCBW1-A and HCBW1-B. In addition, the displacements corresponding to their maximum resistances were relatively similar, considering that the one of Wall HCBW1-A was only a 20.6% higher than that of Wall HCBW6. On the other hand, a softer resistance drop can be observed in Wall HCBW6, which exhibited a reduction of 20% in its maximum lateral resistance at a drift 75.0% higher than the drift at maximum resistance ($\Delta_{Vmax}$). That lateral force reduction was observed at a drift considerably large in comparison with the corresponding to Walls HCBW1-A and HCBW1-B (20.6% and 37.6%, respectively). It seems that the transversal elements do not contribute to the in-plane lateral shear strength (of the main panel), principally due to lateral deformations corresponding to the maximum resistance are not large enough to engage an out-of-plane resistant mechanism in the edge elements. Therefore, the out-of-plane elements should be excluded in the calculation of the in-plane shear resistance (in the longer direction) (the discussion on this issue is extended in Section 5). However, edge elements formed a confining frame that provides integrity to the wall when the damage is extensive, which considerably improved the seismic behavior.

Finally, Figure 7f compares the envelope curves of Walls HCBW1-A and HCBW1-B, and HCBW7 to illustrate the effect of the wall aspect ratio. The shear strength of the HCBW7 wall was approximately 25% lower regarding the reference walls. However, this shear strength was reached at a higher deformation. Besides, while the reference walls presented a strong decay in their post-peak shear strength, the HCBW7 wall maintained its peak strength for more cycles beyond the maximum. Therefore, increasing the wall aspect ratio resulted in a higher contribution of the flexural deformation mode and less brittle behavior.

### 3.2. Damage Progression

**Figure 8:**
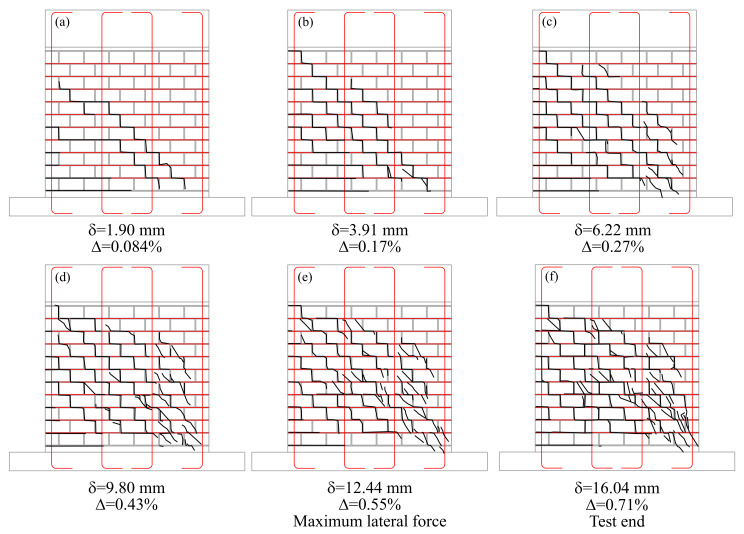
Damage on the wall HCBW1-A at different lateral displacement levels: (**a**) 1.90 mm, (**b**) 3.91 mm, (**c**) 6.22 mm, (**d**) 9.80 mm, (**e**) 12.44 mm, and (**f**) 16.04 mm.

The damage progression on Wall HCBW1-A was tracked by mapping the cracks at the peak displacement in the push loading direction of different load cycles, as presented in Figure 8. The first crack map (Figure 8a) was generated when the first major diagonal crack was detected, at a lateral displacement (δ) of 1.90 mm. In Figure 8a, some horizontal cracks can also be identified in the first mortar bed-joints of the left edge, which were a result of the moment induced by the external displacement that was precisely highest at the base of the masonry panel. For this reason, those cracks are named as horizontal flexural cracks hereinafter. Then, new cracks appeared, mainly in block–mortar interfaces, as lateral deformation was incremented. Those cracks formed a system of stair-step cracks, as can be observed in Figure 8b,c. These crack patterns indicate that block–mortar interfaces made up a plane of weakness because their bond strength was lower than the tensile strength of HCBs. In this regard, the presence of horizontal reinforcement was fundamental to force other damage mechanisms to take place. Despite the generation of new cracks and propagation of the existent ones, the BJR was able to keep masonry tied after cracking, as also reported by Tomaževič and Lutman [26] and Schultz et al. [22]. Afterward, stair-stepped cracks also grew through blocks to form distinguishable diagonal cracks, a process that started from the toe under compression (bottom-right zone), as shown in Figure 8d–f. By the end of the test (Figure 8f), the cracking of blocks in the bottom right zone was severe enough to generate a decay in the lateral resistance. It also can be observed that the presence of interior vertical reinforcement bars did not deviate the crack pattern from the characteristic diagonal crack pattern of shear-dominated failures. This situation is contrary to the observed in studies carried out in shear walls made of multi-perforated clay bricks [35] and hollow clay blocks [36].

**Figure 9:**
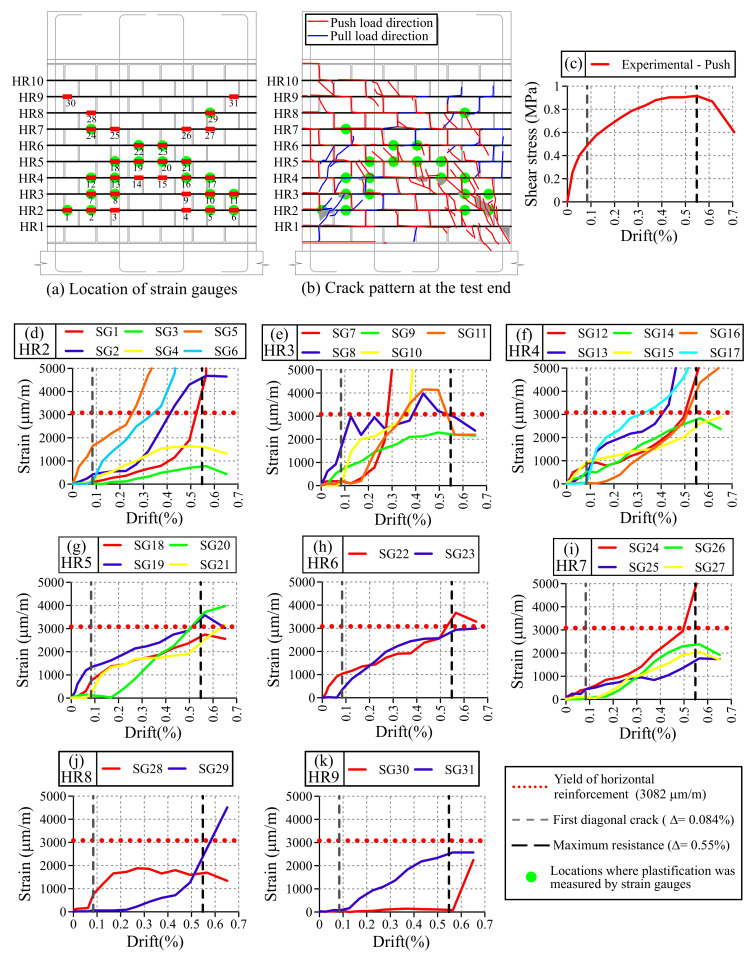
Strain gauge schemes and measurements for the wall HCBW1-A: (**a**) location of strain gauges, (**b**) crack pattern at the test end, (**c**) envelope curve in the push load direction; Envelope curves of the strain gauges in the push load direction at: (**d**) HR2, (**e**) HR3, (**f**) HR4, (**g**) HR5, (**h**) HR6, (**i**) HR7, (**j**) HR8, and (**k**) HR9.

The progression of damage on Wall HCBW1A was also monitored with several strain gauges (SGs), which were stuck to horizontal reinforcement elements at the locations indicated in Figure 9a. Thirty-one SGs were employed in total, distributed among horizontal reinforcements HR2–HR9 (codification at the left edge of Figure 9a), in an arrangement that followed zones likely to crack. Figure 9b shows the crack maps developed at the end of the tests, where cracks generated in both loading directions are superimposed. Additionally, Figure 9a,b indicates with green circles the locations where the measured strains exceeded the yield strain of horizontal reinforcement steel ($\varepsilon_{y,HR}\;=\;3082\;\mu$m/m). Figure 9d–k present the records of the SGs at the maximum deformation of first cycles of each drift increment only in the push direction, where each graph includes the SGs located at the same horizontal reinforcement element. These plots also indicate the yield strain of the horizontal reinforcement steel ($\varepsilon_{y,HR}\;=\;3082\;\mu$m/m) as a horizontal red dotted line, and the drifts of formation of the first diagonal crack ($\Delta_{1cr}$=0.084%) and maximum resistance in the push loading direction ($\Delta_{Vmax}$=0.55%) with vertical dashed lines in gray and black, respectively. As only results in the push direction are plotted, Figure 9c presents the corresponding experimental envelope curve. It is important to remark that the push loading direction was selected due to maximum lateral resistance was achieved in that direction, and only the first cycle is presented to improve clarity.

As can be observed in Figure 9d–k, horizontal rebars presented deformations before the first diagonal crack was visually detected on walls. Considering that reinforcement starts working once cracking has taken place [26], Wall HCBW1A could have experienced cracking before it was noticed in the visual inspection. Nonetheless, the SGs that firstly recorded strains (SGs 5, 8, 12, 19, 22, 24, and 28) in each reinforcement element were mainly nearby the crack mapped at a drift (Δ) of 0.084% (δ = 1.90 mm, Figure 8a), which confirms the location of the first identified stair-stepped diagonal crack. Moreover, those SGs indicate that a compression strut should exist in the diagonal band where they were located.

Additionally, the low strains measured by SGs 1, 2, 3, and 7 (Figure 9d,e) validate the hypothesis that horizontal cracks at the left toe were because of bending, since no axial strain was imposed on rebars because the block–mortar joint unstuck. In this regard, the formation of those horizontal cracks was necessary to engage the vertical reinforcement rebars, following the same principle of passive reinforcement as horizontal rebars.

On the other hand, many SGs started measuring strains once the drift surmounted 0.084% (DS4, visual detection of the first major diagonal crack in the push loading direction), mainly the SGs located on the compressed diagonal (diagonal from top left to bottom right corners of the panel). In this process, the first SG that recorded a strain higher than the yield strength of horizontal rebars ($\varepsilon_{y,HR}$) was the SG5 at a drift (Δ) equal to 0.27% (δ = 0.73 mm). Subsequently, a redistribution on the deformation pattern occurred because other SGs started recording strains, as can be noticed in Figure 9d–k. This phenomenon suggests that, once the horizontal rebars plasticize in a location, deformation pattern redistributes in order to keep gaining lateral resistance.

Afterward and before reaching the deformation at maximum lateral force, other SGs also exceeded the yield strain of the shear reinforcement ($\varepsilon_{y,HR}$), in particular SGs 7, 6, 10, 11, 17, 2, 8, 13, 1, 12, 16, 19, 20, 22, and 24 (sorted according the plasticization sequence). These SGs were not all on the compressed diagonal of the wall, but mainly in the zone below that diagonal, at the locations where cracks were mapped when maximum lateral force was recorded (Figure 8e). Finally, once the wall was losing resistance, strains continued growing in most SGs, especially in those located in the inferior half of the panel. This behavior was mainly due to the extensive damage at both wall toes, which was generated by the compression struts that acted in both load directions, as illustrated in Figure 9b.

**Figure 10:**
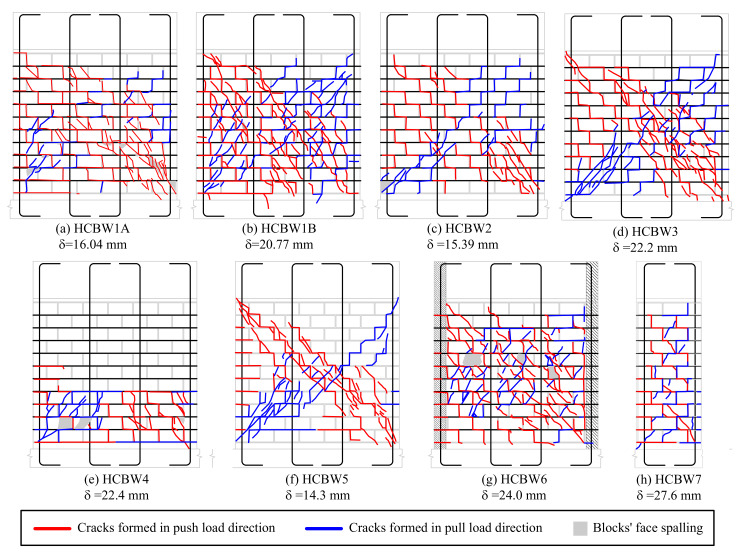
Crack patterns observed at the end of the tests on the walls: (**a**) HCBW1-A, (**b**) HCBW1-B, (**c**) HCBW2, (**d**) HCBW3, (**e**) HCBW4, (**f**) HCBW5, (**g**) HCBW6, and (**h**) HCBW7.

Figure 10 presents the crack patterns observed in the tested walls at the end of the tests. The base walls (Figure 10a,b) exhibited a very similar distribution of cracks, although Wall HCBW1-B was more cracked since it was subjected to a higher lateral deformation. In general, cracks went through HCBs and block–mortar interfaces, where it was not possible to appreciate that grouted zones limited the propagation of cracks. The propagation of cracks was generally diagonal, and in a group, the cracks that arose in the same loading direction tend to be aligned with the downward diagonal according to the direction of lateral displacement. The group of diagonal cracks suggests the position of the compression strut in each load direction because both should be aligned and at the same location. However, the width of the compression strut cannot be identified in Figure 10a,b because crack patterns have notorious differences. Some inclined cracks can also be noticed below the diagonal crack bands in both loading directions. Those inclined cracks appeared in advance stages of damage, unlike the horizontal flexural cracks that were formed at the beginning of the test and whose widths remained stable through the tests.

Wall HCBW2 exhibited a crack map with less cracked blocks than the base walls (Figure 10c), except in its compressed toes. Using blocks with higher tensile strength incremented the possibility of block–mortar interfaces to be the weakest link zone because block-to-mortar bond strength did not increase when using stronger blocks, as previously commented. Therefore, variations in the crack pattern were a consequence of the higher strength of the HCBs employed in Wall HCBW2.

On the other hand, Wall HCBW3 exhibited a crack pattern (Figure 10d) very similar to the base Wall HCBW1-A, although it achieved a lateral displacement comparable with Wall HCBW1-B. This situation implies that increasing the width of the blocks decreases the extension of cracks, which provides integrity to the wall in the post-peak regime, as previously discussed.

Figure 10e shows that reducing the vertical reinforcement ratio ($\rho_{v}$) generated a drastic variation on the crack pattern, where damage concentrated in the first four block courses of Wall HCBW4. The lower flexural stiffness allowed a higher rotation of the panel, with a crack pattern that recalls a plastic hinge. Note that plastic hinges are desired when designing ductile shear walls. Although Wall HCBW4 had a lower shear strength than the reference walls, the concentrated crack pattern might be preferable for repairing purposes because a smaller zone of the wall would require restoration or retrofit.

On the other hand, removing the shear reinforcement also modified the crack pattern, as can be noticed in Figure 10f. Wall HCBW5 showed a more delimited diagonal crack band than the reference walls, which is a sign of the capability of horizontal steel elements to force crack spreading throughout the panel. Providing shear reinforcement also increases the ratio of the total area of the panel that is effectively employed to resist lateral forces, and, therefore, the efficiency of the construction system. In this case, the presence of vertical rebars and grouted cells did not have a remarkable effect on the crack pattern since the trajectory of cracks was not altered by those elements, as already observed in Walls HCBW1-A and HCBW1-B.

The wall with edge elements (HCBW6, Figure 10g) concentrated the diagonal cracking in the interior of the panel, a zone that presented a similar crack pattern as the reference walls. The toes of Wall HCBW6 were not crushed or extensively cracked because of the higher cross-section at the edges. In fact, only horizontal flexural cracks were noticed in the transversal elements, which were needed by vertical reinforcement to work.

Finally, Figure 10h shows the crack pattern of Wall HCBW7. This wall presented horizontal cracks at the bottom of the grouted cores, because of the contribution of a flexural deformation mode. Diagonal cracks were observed through the entire height of the wall, running along the mortar joints, both as stair-stepped cracks and through the blocks. The inclination of these cracks was similar to the observed in the other walls. However, it is not possible to identify a diagonal crack band or the compression struts because of the slender geometry. Moreover, no significant spalling was observed during the test.

## 4. Assessment of Seismic Demand Parameters

### 4.1. Shear Strength

**Figure 11:**
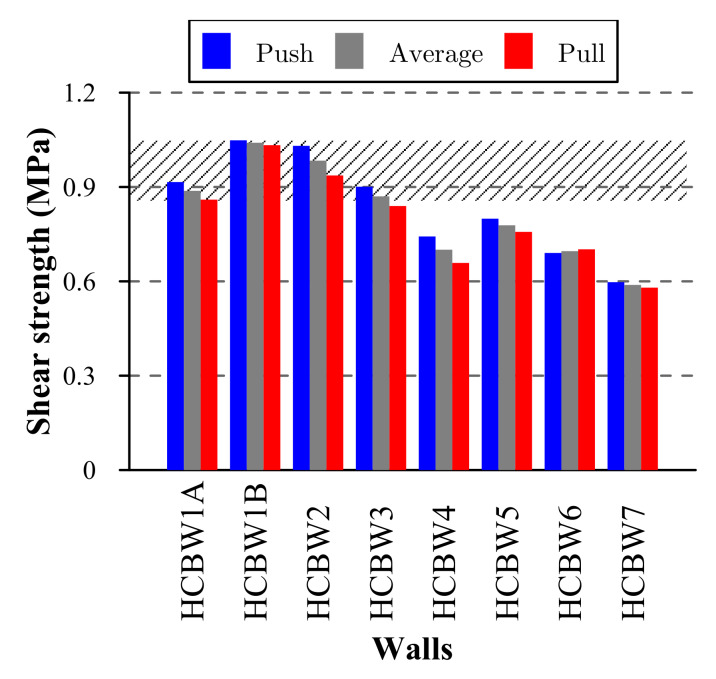
Loading direction effects on the shear strength.

Figure 11 compares the shear strength measured in the performed tests, where a lower shear strength can be noticed in all walls in the pull loading direction, except for Wall HCBW6 that had almost the same resistance in both load directions. In this regard, the edge elements of Wall HCBW6 provided integrity and symmetry to the response.

The dashed area in Figure 11 represents the range of shear strengths that might be expected on walls constructed following the design specifications employed for building the reference walls. The upper limit is the shear strength of Wall HCBW1-B in the push loading direction ($\tau_{max}^{Push}$ = 1.05 MPa) and the lower limit is the shear strength of Wall HCBW1-A in the pull loading direction ($\tau_{max}^{Pull}$ = 0.86 MPa).

As can be appreciated, only Walls HCBW2 and HCBW3, those built with stronger and thicker blocks, respectively, exhibited similar resistances to the reference walls. The failures in the reference walls (HCBW1-A and HCBW1-B) and the modified (HCBW2) wall were concentrated at blocks-mortar interfaces, rather than blocks. For this reason, the effect on the shear strength related to the use of a stronger block was unnoticed. Indeed, an increment in the shear strength would have been observed if a higher block–mortar bond strength was provided by modifying any design parameter.

On the other hand, a reduction in the vertical (HCBW4) or horizontal (HCBW5) reinforcement ratio produced a reduction in the shear strength. These effects were already reported by Sierra [23] and the authors of [14,16,21], respectively.

The presence of flanges reduced the shear resistance because they incremented the cross-section area but did not effectively contribute to the lateral resistance, as commented. Nonetheless, if the area of out-of-plane elements is neglected, the shear strength ($\tau_{max}^{Push}$ = 1.03 MPa, $\tau_{max}^{Pull}$ = 1.04 MPa, and ${\bar{\tau }}_{max}$ = 1.04 MPa) was within the range delimited by the strength of the reference walls. In this regard, the presence of edge elements only affected other indicators of seismic performance. Finally, a higher wall aspect ratio (Wall HCBW7) led to a reduction in the shear strength of the wall, as also indicated by Voon and Ingham [4] and Ramírez et al. [21].

### 4.2. Degradation of Lateral Stiffness

The degradation of the secant stiffness of the elements was monitored as the ratio between the secant stiffness of a cycle *i* ($K_{sec,i}$) and the initial lateral stiffness ($K_{sec,0}$) of the element. $K_{sec,i}$ can be calculated with Equation (2), where $V_{max,i}^{+}$ and $V_{min,i}^{-}$ are the maximum and minimum loads of the cycle *i* and $\delta_{max,i}^{+}$ and $\delta_{min,i}^{-}$ are the maximum and minimum deformations of the cycle *i*, respectively. This measurement can be useful to assess the influence of design properties on the performance of a wall under successive cyclic displacements, which is of particular interest in seismic-prone locations.

(2) $$K_{sec,i}=\frac{V_{max,i}^{+}-V_{min,i}^{-}}{\delta_{max,i}^{+}-\delta_{min,i}^{-}}$$

**Figure 12:**
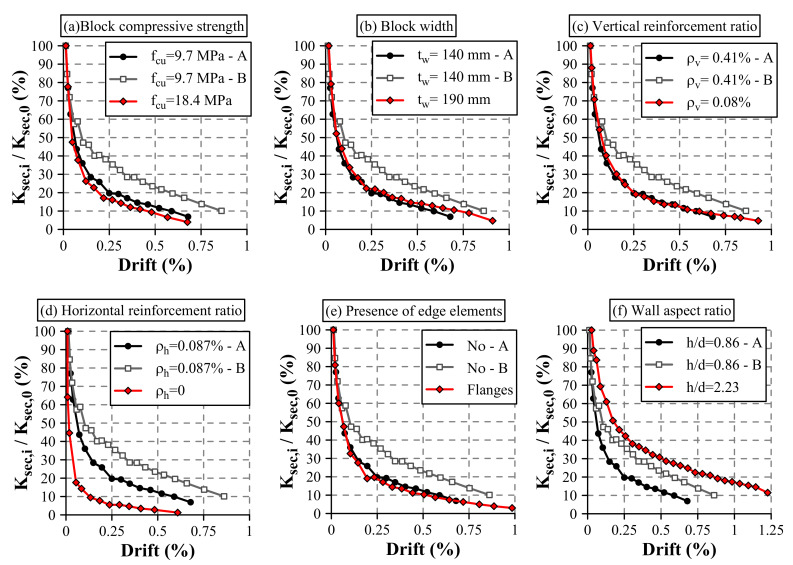
Influence of design parameters on the lateral stiffness degradation: (**a**) block compressive strength, (**b**) block width, (**c**) vertical reinforcement ratio, (**d**) horizontal reinforcement ratio, (**e**) presence of edge elements, and (**f**) wall aspect ratio.

Figure 12 presents the evolution of the ratio $K_{sec,i}/K_{sec,0}$ as a function of the drift ratio, where reference Walls HCBW1-A and HCBW1-B are, respectively, presented with black and grey lines in all subplots. In general terms, the walls showed a rapid stiffness decay up to a drift of 0.15% approximately, followed by a stiffness deterioration with constant rate roughly. It can also be observed that Wall HCBW7 presented the softest degradation among tested walls (Figure 12f). The high wall aspect ratio of this specimen produced a combination of shear and flexural failure modes, which caused a less brittle behavior.

Among the specimens with the same wall aspect ratio, Wall HCBW1-B presented the softest stiffness degradation. The fastest degradation was observed in the wall without horizontal reinforcement (HCBW5) (Figure 12d). In fact, Walls HCBW1-A, HCBW2, HCBW3, HCBW4, and HCBW6 presented very similar secant lateral stiffness decay shapes, losing 50% of their initial stiffness ($K_{sec,0}$) at a drift of 0.07%, approximately. The insensibility of the stiffness degradation rate to the vertical reinforcement amount has already been indicated by Sierra [23]. Accordingly, the horizontal reinforcement ratio seems to be the one studied design variable that modifies the evolution of the stiffness degradation since it enhances the ability of distributed bed-joint reinforcement to provide post-cracking integrity to the masonry compound.

### 4.3. Equivalent Viscous Damping Ratio

Equivalent viscous damping ratio ($\xi_{eq}$) can be used to study the capacity of energy dissipation of a construction system. Particularly, in alternated loading tests, $\xi_{eq}$ can be calculated for every load cycle *i* ($\xi_{eq,i}$) using Equation (3), where $E_{s,i}$ is the energy dissipated in the cycle *i* (area enclosed by the hysteresis curve along a complete cycle), $K_{sec,i}$ is the secant stiffness corresponding of the cycle *i* (Equation (2)), and $\delta_{max,i}$ is the maximum lateral displacement of the cycle *i*. It is worth noticing that this indicator of energy dissipation capacity is normalized with respect to the elastic energy that an equivalent elastic system should develop for a given lateral deformation.

(3) $$\xi_{eq,i}=\frac{E_{s,i}}{2\cdot \pi \cdot K_{sec,i}\cdot \delta_{max,i}^{2}}$$

**Figure 13:**
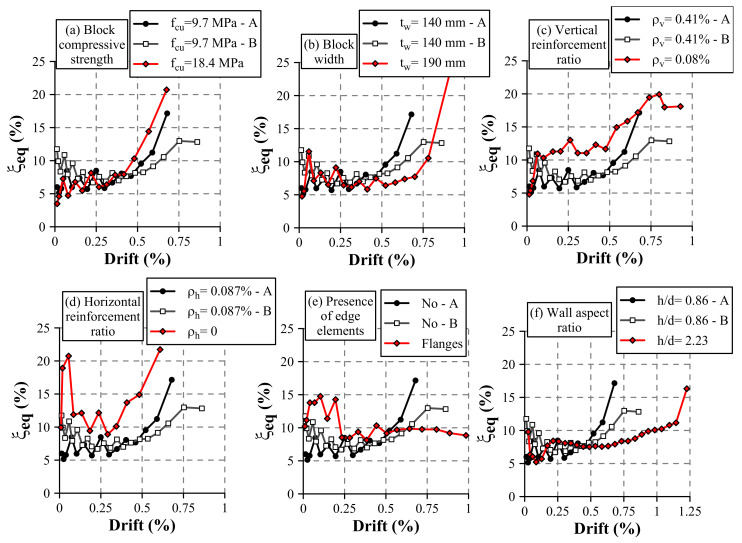
Effect of design parameters on the equivalent viscous damping: (**a**) block compressive strength, (**b**) block width, (**c**) vertical reinforcement ratio, (**d**) horizontal reinforcement ratio, (**e**) presence of edge elements, and (**f**) wall aspect ratio.

Figure 13 presents the evolution of this parameter as a function of the drift. As in previous figures, the reference Walls HCBW1-A and HCBW1-B are presented in all subplots with black and grey lines, respectively. It can be observed that Wall HCBW1-A presented an incremental profile that starts at a damping ratio equal to 5%. On the other hand, Wall HCBW1-B presented a higher damping ratio at the beginning and the end of the test and showed a plateau about a damping ratio of 7.5% for drifts between 0.15% and 0.5%. Even though Wall HCBW1-B started with a higher viscous damping ratio than Wall HCBW1-A, this relation was inverse at the end of the tests.

In comparison with the reference walls, incrementing the strength of the blocks did not have a significant influence until reaching the last stage of the tests, where a higher viscous damping ratio was shown by Wall HCBW2 (Figure 13a). A similar trend can be observed when analyzing walls with different width (Figure 13b), where no apparent effect could be distinguished at the beginning of the tests. However, at the end of the test, the wall with thicker blocks exhibited a significant increment in its viscous damping ratio. Therefore, improving the resistance or the width of the block increases the energy dissipation capacity for a severe damage stage.

The walls with a reduced amount of vertical and horizontal reinforcement exhibited a higher capacity of energy dissipation in general, as appreciated in Figure 13c,d, respectively. In this figure, a similar equivalent viscous damping can be observed for all walls at smaller drift values, but Walls HCBW4 and HCBW5 showed a rapid increment in the equivalent viscous damping when drift values increased. Furthermore, the wall without horizontal reinforcement (HCBW5) exhibited a sudden increment on the equivalent viscous damping ratio at drifts between 0.02% and 0.05%, which can be explained by the development of several shear cracks at the beginning of the test.

On the other hand, the wall with flanges (Figure 13e) exhibited an initial equivalent viscous damping ratio similar to the values of the reference walls. Nonetheless, that value rose to an approximate value of 14% for drifts between 0.04% and 0.2%, and, subsequently, it remained constant for drifts beyond 10% approximately. The stable equivalent viscous damping ratio value exhibited by this latter wall through different damage states indicates the stability and confinement that were provided by the transversal elements.

Regarding the wall aspect ratio, Wall HCBW7 started with a similar $\xi_{eq}$ value as Wall HCBW1-B but plummeted at a drift of 0.04%. From drift values of 0.2% to 0.8%, the equivalent damping ratio remained stable and then started increasing until the end of the test.

It is important to mention that, although Walls HCBW4, HCBW5, and HCBW6 exhibited a higher equivalent viscous damping ratio than the reference walls, it is not necessarily correct to conclude that such walls were capable of dissipating more energy in absolute terms. This is because the equivalent viscous damping ratio ($\xi_{eq}$) is an index that is normalized by the exhibited secant stiffness ($K_{sec}$). Therefore, a low secant stiffness could result in high values of equivalent viscous damping ratio ($\xi_{eq}$). Finally, it is important to remark that, independently of the observed differences, all walls presented an equivalent viscous damping ratio between 8.1% and 14.9% for the intermediate test stages (i.e., drifts approximately between 0.2% and 0.5%). These values are slightly over the range of values reported by Ramírez et al. [21] ($\xi_{eq}$ between 5% and 11%) for a comparable damage level.

### 4.4. Ductility

Several authors (e.g., [37,38,39,40]) have proposed different methodologies for calculating the displacement ductility of a shear wall, an indicator of the capacity of an element to incur in the inelastic range without experiencing a catastrophic loss of resistance. Most approaches idealize the envelope curve with a bilinear curve and then calculate the ductility as the ratio among the deformation associated with the end of the idealized elastic behavior (or the yielding of the element) ($\delta_{y}$) and the displacement of ultimate resistance ($\delta_{u}$). Although the idealized bilinear curve is often calculated to enclose the same energy as the envelope curve up to $\delta_{u}$, there is no consensus on how to determine $\delta_{y}$ or $\delta_{u}$. In this regard, there is evidence that employing different methods could lead to completely different results [38]. Moreover, some approaches [37,40] require identifying when the envelope curve has a drastic variation on the tangent stiffness, and this process might be subjective.

**Figure 14:**
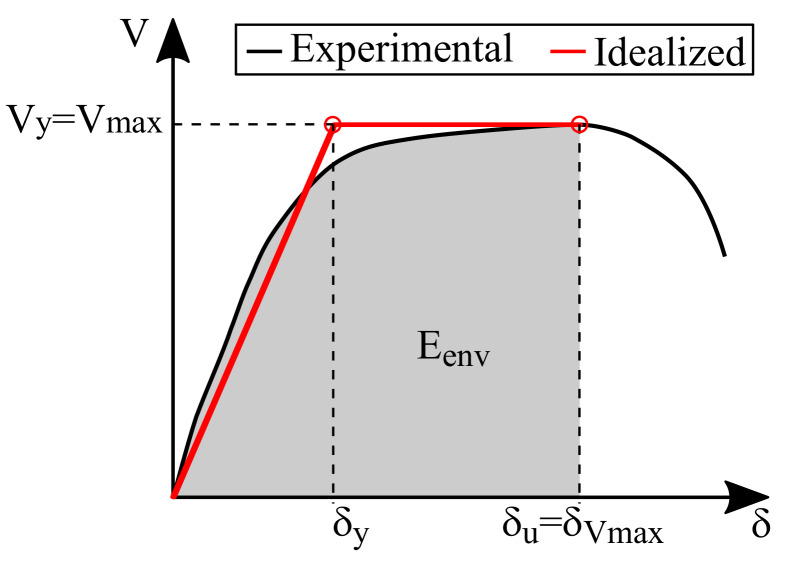
Idealization of the envelope curve.

Considering this context, the idealized envelope curve and the ductility in a load direction were calculated herein with a conservative approach. Consequently, $\delta_{u}$ was assumed as the deformation at maximum lateral resistance ($\delta_{Vmax}$), as already done in previous studies [21,28,35,37,41]. This hypothesis is based on the fact that the post-peak response of masonry shear walls exhibits large variability and represents an undesirable damage state to achieve. Thus, the post-peak deformation capacity was neglected. It was also assumed that the idealized and experimental envelope curves must present the same energy up to $\delta_{u}$. Thus, the lowest ductility ($\mu_{d}$) is obtained when the yield force is the same as the wall’s resistance ($V_{max}$), which turns the idealized curve into an elastoplastic bilinear, as shown in Figure 14. Based on these assumptions, the displacement ductility of a wall can be calculated with Equation (4), where $E_{env}$ is the area below the envelope curve in the analyzed direction up to $\delta_{Vmax}$.

(4) $$\mu_{d}=\frac{\delta_{u}}{\delta_{y}}=\frac{\delta_{Vmax}}{2\cdot \left(\delta_{Vmax}-\frac{E_{env}}{V_{max}}\right)}$$

**Figure 15:**
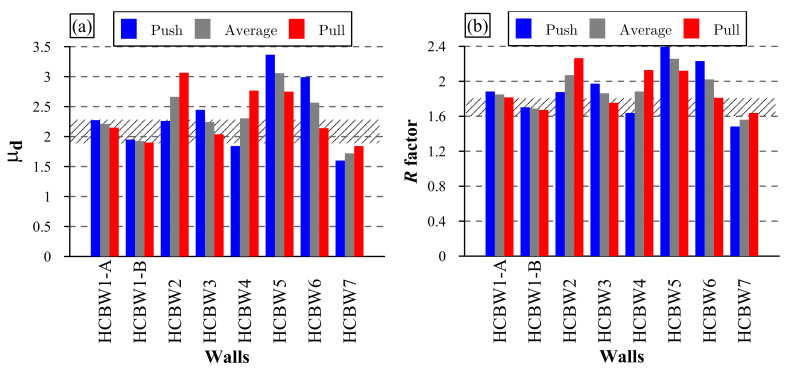
(**a**) Displacement ductility; and (**b**) seismic force-reduction (R) factor.

The parameters of the idealized envelopes and the ductility of the tested walls are presented in Table 5. Besides, Figure 15a compares the ductility values calculated in both load directions and on average. As can be observed, the ductility values ranged between 1.60 and 3.37. Moreover, most of the walls exhibited a higher ductility in the push loading direction, except for Walls HCBW2, HCBW4, and HCBW7. As expected, the obtained ductility values were considerably lower than the values reported by Ramírez et al. [21] (reported values from 2.85 to 7.95) because the calculation methodology adopted in this research is conservative. On the other hand, the base Walls HCBW1-A and HCBW1-B had average ductility values ($\bar{\mu_{d}}$) (between both loading directions) equal to 2.21 and 1.93, respectively. These results exemplify the fact that a wall with higher lateral resistance does not necessarily have a higher displacement ductility.

The dashed area in Figure 15a shows the range of ductility values observed in the reference walls, whose lower and upper bounds were 1.90 (pull loading direction of Wall HCBW1-B) and 1.88 (push load direction of Wall HCBW1-A), respectively. Although the average ductility ($\bar{\mu_{d}}$) of Wall HCBW2 was higher than the observed on the base walls, the value in the push loading direction was in the dashed area, which indicates that the effect was not significant enough.

Similarly, the wall with transversal elements (HCBW6) presented an average ductility ($\bar{\mu_{d}}$) higher than the reference walls, but the value in the pull loading direction was not higher enough. Therefore, further evidence must be collected to validate these possible effects.

Regarding Walls HCBW3 (thicker block) and HCBW4 (lower vertical reinforcement ratio), there was no apparent influence on the ductility. In both walls, the ductility in one load direction is outside the dashed area (push load direction for Wall HCBW3 and pull load direction for Wall HCBW4) (Figure 15a), but the average ductility is inside the dashed area.

Contrary to the expected, the horizontally unreinforced wall exhibited the highest ductility values. Nonetheless, this situation is a consequence of the lower yield deformation, which is associated with the cracks reported at low deformations at the beginning of the test.

Even though Wall HCBW7 did not exhibit a sudden post-peak degradation as with the other walls, it had the lowest ductility values. In the case of Wall HCBW7, neglecting the post-peak resistant capacity when calculating the ductility is over-conservative.

The seismic-force reduction factor (*R*) of tested walls was calculated with the equation proposed by Paulay and Priestly [42] for buildings of a short period (Equation (5)). The obtained values are included in Table 5 and presented in Figure 15b, where it can be observed that R-factor ranged from 1.48 to 2.39. It is worth mentioning that the same trends noticed for ductility ($\mu_{d}$) can be noticed for the R-factor due to the form of Equation (5). It is also important to highlight that the average R-factor among tested walls in both load directions, excluding Wall HCBW5 that is not strictly a reinforced masonry shear wall, is equal to 1.85 (CV = 11.2%). This value is 38.4% lower than 3.0, the R-factor indicated by the Chilean seismic design code for buildings of reinforced masonry of HCB [43]. One cause of the difference regarding the normative R-factor values might be the fact that ductility values were calculated by neglecting the post-peak deformation capacity and assuming a conservative approach.

(5) $$R=\sqrt{2\mu_{d}-1}$$

An appropriate criterion to measure the ductility of PG-RM shear walls must be stated. It is essential to propose a ductility calculation methodology that will be independent of the judgment and appreciation of who processes the information. It is also highlighted that the variability associated with the post-peak response complicates relying on the wall post-peak deformation capacity, as has been commented throughout this research article. Moreover, it has been statistically observed that the shear failure of PG-RM shear walls is more likely a force-controlled phenomenon than a deformation-dominated phenomenon [10]. Furthermore, ductility calculations affect the estimation of R-factors, and, thus, the safety factors provided to the buildings. Therefore, this issue rises as an urgent need.

## 5. Estimation of Shear Resistance

Several studies have proven that existent design expressions are not accurate enough (e.g., [5,7,8,9]), although they have mainly focused on bond-beam reinforced masonry shear walls. Some investigations [6,21] have evaluated the accuracy of selected expressions to predict the lateral resistance of bed-joint reinforced shear walls, but the evidence is limited. Therefore, the expressions proposed by Shing et al. [3] (Equation (6)) and Psilla and Tassios [44] (Equations (7)–(9)), as well as the code expressions CSA-S304 [45] (Equations (10)–(14)) and TMS402/602 [46] (Equations (15)–(18)), were evaluated. It is worth noting that, to improve the readability of the text, the meaning of symbols employed in Equations (6)–(18) is presented in the Notation List. Additionally, $k_{bh}$ and $k_{bv}$ in Psilla and Tassios’ expression [44] (Equations (7)–(9)), factors which represent the type of anchorage of reinforcing bars, are taken as 3.0 and 2.0, respectively, due to the characteristics of tested walls.

(6) $$\begin{array}{c} V_{n1}=\left(0.166+0.0217\rho_{v}f_{yv}\right)A_{n}\sqrt{f_{m}^{\prime }}+0.0217\sigma_{n}A_{n}\sqrt{f_{m}^{\prime }}+\left(\frac{L_{w}-2d^{\prime }}{s_{h}}-1\right)A_{sh}f_{yh}\; \end{array}$$

(7) $$\begin{array}{c} V_{n2}=\sqrt{\frac{Vd_{v}}{M}}\cdot \left(0.03\sqrt{f_{m}^{\prime }}L_{w}t+0.3P\right)+\left(\frac{2}{3}\lambda_{h}A_{sht}f_{yh}+0.2\lambda_{v}A_{svt}f_{yv}\right) \end{array}$$

(8) $$\begin{array}{c} \;\;\lambda_{h}=1-\frac{0.6}{k_{bh}}\cdot \frac{f_{yh}}{f_{tm}}\cdot \frac{d_{sh}}{L_{w}}\geq 0 \end{array}$$

(9) $$\begin{array}{c} \;\;\lambda_{v}=1-\frac{0.6}{k_{bv}}\cdot \frac{f_{yv}}{f_{tg}}\cdot \frac{d_{sv}}{h_{w}}\geq 0 \end{array}$$

(10) $$\begin{array}{c} V_{n3}=\left(\tau_{m}td_{v}+0.25P\right)\cdot \gamma_{g}+0.6A_{sh}f_{yh}d_{v}/s_{h}\leq V_{n3,max} \end{array}$$

(11) $$\begin{array}{c} \;\;\tau_{m}=0.16\left(2-\frac{M}{V\cdot d_{v}}\right)\sqrt{f_{m}^{\prime }}\leq 0.4\sqrt{f_{m}^{\prime }}A_{n} \end{array}$$

(12) $$\begin{array}{c} \;\;0.25\leq M/(V\cdot d_{v})\leq 1.0 \end{array}$$

(13) $$\begin{array}{c} \;\;V_{n3,max}=\left\{\begin{array}{cc} 0.4\sqrt{f_{m}^{\prime }}td_{v}\gamma_{g} & ,\;\mathrm{if}\;h_{w}/L_{w}>1.0\\ 0.4\sqrt{f_{m}^{\prime }}td_{v}\gamma_{g}\left(2-h_{w}/L_{w}\right) & ,\;\mathrm{if}\;0.5<h_{w}/L_{w}\leq 1.0\\ 0.4\sqrt{f_{m}^{\prime }}td_{v}\gamma_{g}\cdot 1.5 & ,\;\mathrm{otherwise}\; \end{array}\right. \end{array}$$

(14) $$\begin{array}{c} \;\;\gamma_{g}=A_{n}/A_{T}\leq 0.5\;\mathrm{for}\;\mathrm{PG}\;\mathrm{walls} \end{array}$$

(15) $$\begin{array}{c} V_{n4}=\gamma_{g}\cdot \left(V_{nm}+V_{ns}\right)\leq \gamma_{g}\cdot V_{n4,max},\;\gamma_{g}=0.75\;\mathrm{for}\;\mathrm{PG}\;\mathrm{walls}\; \end{array}$$

(16) $$\begin{array}{c} \;\;V_{nm}=0.083\cdot \left(4-1.75M/\left(Vd_{v}\right)\right)A_{n}\sqrt{f_{m}^{\prime }}+0.25P \end{array}$$

(17) $$\begin{array}{c} \;\;V_{ns}=0.5A_{sh}f_{yh}d_{v}/s_{h} \end{array}$$

(18) $$\begin{array}{c} \;\;V_{n4,max}=\left\{\begin{array}{cc} 0.5A_{n}\sqrt{f_{m}^{\prime }} & ,\;\mathrm{if}\;M/(Vd_{v})\leq 0.25\\ \left(0.56-0.23M/\left(Vd_{v}\right)\right)A_{n}\sqrt{f_{m}^{\prime }} & ,\;\mathrm{if}\;0.25<M/(Vd_{v})<1.0\\ 0.33A_{n}\sqrt{f_{m}^{\prime }} & ,\;\mathrm{if}\;M/(Vd_{v})\geq 1.0 \end{array}\right. \end{array}$$

**Figure 16:**
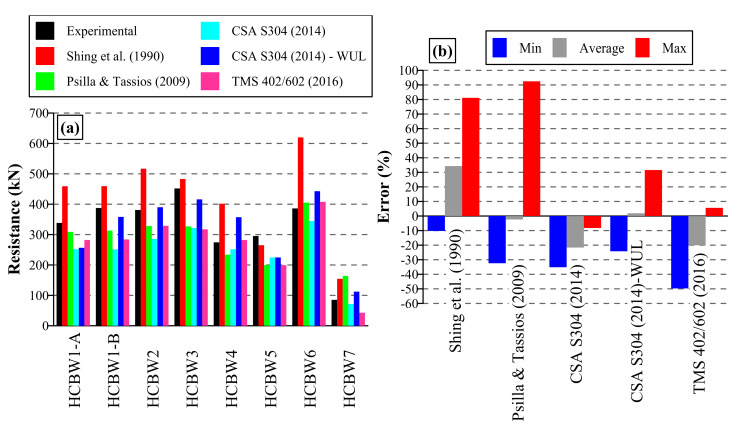
(**a**) Prediction of resistance with different expressions; and (**b**) prediction errors.

The calculated resistances are presented in Table 6 and compared in Figure 16a. It is important to mention that the upper limit of CSA S304’s [45] expression ($V_{n3,max}$, Equation (13)) limited the calculated resistance of all walls, with the exception of the wall without horizontal reinforcement (HCBW5). Therefore, calculations without the upper limit (WUL) were also included in this analysis.

As can be observed, a wide range of predictions can be noticed for each wall among different expressions, and it is not possible to identify an expression that performs acceptably for all walls. Therefore, results were analyzed following the methodology employed in Aguilar et al. [6], with a measurement of error defined as ($\left(V_{n}-V_{exp}^{max}\right)/V_{exp}^{max}$). This index takes a positive value when the prediction is an overestimation and a negative value when it is a sub-estimation. Table 7 includes average, maximum, and minimum values and the standard deviation of this error index, which are also illustrated in Figure 16b. As can be observed, none of the analyzed expressions were accurate enough and a great range of predictions was obtained (the difference between the minimum and maximum errors). In fact, the best expression on average was the CSA S304 [45] without considering the upper limit, with an average error of 1.9%, which is slightly non-conservative. Nonetheless, this expression was associated with a wide range of values that fluctuates from −21.7% to 34.3%, similar to the other evaluated expressions.

As commented above, out-of-plane elements had a slight contribution to the lateral resistance of the wall in the long direction and that they should be excluded in the estimation of the shear resistance. Indeed, if the out-of-plane sections are neglected, the design of Wall HCBW6 resembles the design of Walls HCBW1 (the only difference is the diameter of the vertical rebars at the edges of the panels). Therefore, if the shear expressions presented in Equations (6)–(18) are used to evaluate the resistance of Wall HCBW6 without the out-of-plane sections, the same values as for Walls HCBW1 are obtained (Table 6). Consequently, if these corrected resistance estimations of Wall HCBW6 are compared with the experimental result of the wall, an average prediction error of −19.3% and a range of errors (the difference between the maximum and minimum prediction errors) of 53.6% is obtained. These values are conservative in comparison to the uncorrected values reported in Table 6 (average error of 15.0%, range of 71%). Therefore, it seems that ignoring the contribution of out-of-plane elements when designing gives conservative results.

It is also important to mention that the evaluated shear expressions estimate the shear strength capacity of the masonry compound as a function of its compressive strength. In these expressions, the estimator takes the form of the square root of the masonry compressive strength, and it is modified by some coefficients (mainly empirically adjusted), as can be observed in Equations (6), (7), (11) and (16). The compressive strength is typically derived from stack-bond prisms compression tests. Nonetheless, a weak correlation between the estimator based on the compressive strength and the shear strength measured in diagonal compression tests have been found [5,8,47]. Therefore, the accuracy of the evaluated shear expressions might be increased by using an estimator based on shear strength derived from diagonal compression tests. Nonetheless, this would create practical issues. Carrying out diagonal compression tests on masonry panels is much complex than compression tests on stack-bond prisms. As a result, when designing, practitioners would prefer to adopt conservative masonry shear strength values than sampling the masonry compound with experimental methods. Therefore, the adoption of a more accurate estimator for the shear strength might also be detrimental from a practical point of view.

## 6. Conclusions

Eight hollow-concrete blocks (HCB) partially-grouted reinforced masonry (PG-RM) shear walls were tested to provide new experimental information. Two of those walls were employed as reference cases. The design parameters of the other six walls were varied to allow studying the influence of the strength and width of blocks, the horizontal and vertical reinforcement ratio, the wall aspect ratio, and the presence of out-of-plane edge elements (flanges). Bed-joint embedded ladder-type horizontal reinforcement was employed. All walls were tested under constant axial load and cyclic imposed displacements, with a cantilever-like boundary condition. The results were analyzed in terms of the failure mode, damage progression, shear strength, degradation of lateral stiffness, equivalent viscous damping ratio, and displacement ductility. Additionally, the accuracy of selected expressions for estimating the in-plane lateral resistance was analyzed. From the results, the following conclusions are drawn:

- All walls exhibited similar shapes of hysteresis curves in both loading directions, reaching the maximum resistance in the push load direction. At the beginning of the tests, the walls exhibited an elastic response stage, followed by progressive degradation of the lateral stiffness up to developing the maximum resistance. Afterward, a strong decay in the resistance was observed in most walls, except for the wall with transversal edge elements and the wall with the highest wall aspect ratio. High variability was observed in the response of walls in the post-peak stage of the tests.
- The two walls that were tested with the same configuration and built with the same materials presented a similar response. However, the maximum lateral resistances differed in 12.6% and the associated lateral displacement in 6.7%.
- The presence of interior vertical reinforcements does not have a remarkable effect on the crack pattern.
- Employing stronger blocks generates a slight increment in the lateral stiffness of walls and a decrement in the lateral deformation at maximum resistance. Nonetheless, the shear strength is not affected by the block resistance because cracks concentrated in block–mortar interfaces, but block–mortar bond strength did not necessarily increase when using blocks of better quality. Despite this, a lower number of cracks on blocks can be expected.
- Increasing the width of units does not have a remarkable effect on the shear strength or the initial tangential stiffness. However, using thicker blocks can generate softer post-peak strength degradation. This effect is attributed to the greater cross-sectional area that was available to distribute developed stresses.
- Reducing the amount of vertical reinforcement has a drastic effect on the response of walls, resulting in lower shear strength and slower post-peak resistance degradation. Moreover, reducing the amount of vertical reinforcement allows the wall to develop a higher rotation and generates a crack pattern with more cracks in the base. This change in the crack pattern might be of particular interest for reparation purposes. In addition, a flexural failure mechanism would be developed if a low amount of vertical reinforcement were provided.
- The presence of distributed horizontal reinforcement has a crucial effect on the response of PG-RM shear walls. The horizontal reinforcement elements start working once first cracks are formed, and, once engaged, they force damage to distribute through the weakest zones of panels. As a result, the efficiency of the material is higher, shear strength is increased, and post-crack integrity is provided.
- Transversal out-of-plane edge elements give integrity to walls, generating softer resistance degradation and improving the symmetry of the response. Nonetheless, these elements do not effectively contribute to the in-plane lateral resistance of the main wall. This fact is because edge elements are working in their out-of-plane direction undergoing small deformations. Consequently, no variation on the lateral resistance of the wall with edge elements was observed regarding the reference walls. Additionally, the damage was distributed in the inner part of the panel (between edge elements) with a similar crack pattern to the observed in the reference walls.
- A higher aspect ratio decreases the shear strength, causes flexural deformation mode to start contributing to the response of the wall, and modifies the crack pattern. Besides, incrementing the aspect ratio reduces the initial tangent stiffness but generates a more gradual stiffness degradation.
- In general, most walls exhibited an equivalent damping ratio that incremented with the lateral deformation, starting from values between approximately 5% and 13%. Then in the intermediate test stage (drifts approximately between 0.2% and 0.5%), damping ratios ranged from 8.1% to 14.9%. More disperse values were observed at the end of the tests.
- Displacement ductility, calculated after adjusting an equivalent elastoplastic curve, ranged from 1.60 to 3.37. A possible beneficial effect was observed in walls with stronger blocks and the wall with edge elements. Contrary to the expected, the highest ductility corresponded to the wall without horizontal reinforcement. This result was because of the low cracking deformation of that wall.
- Resistance reduction factor (R-factor) of tested walls that had horizontal reinforcement ranged from 1.5 to 2.4 (mean = 1.85, CV = 11.2%). These values are lower than 3.0, the R-factor indicated for this type of elements by the Chilean seismic design code [43].
- None of the evaluated expressions for estimating the lateral response of PG-RM shear walls ([3,44,45,46]) gave appropriate results. The range of predictions was considerable, which exposes the need for proposing expressions or methods with better performance. In this regard, future developments might investigate modifying the estimator of the contribution of the masonry to the lateral resistance, which currently is a function of the masonry compressive strength.

## Figures and Tables

**Table 1:** Summary of wall characteristics.

Wall	Length (mm)	Height (mm)	Block Width (mm)	Block Type	Wall Aspect Ratio	Gross Cross-Section Area	Flanges	Block Compressive Strength $f_{\mathrm{cu}}$ (MPa)	Masonry Compressive Strength $f_{m}^{\prime }$ (MPa)	Axial Load, $\sigma_{n}/f_{m}^{\prime }$ (%)	Horizontal Reinforcement Ratio $\rho_{h}$ (%)	Vertical Reinforcement Ratio $\rho_{v}$ (%)
HCBW1-A	2640	2270	140	G14	0.86	369600	No	9.7	9.67	5	0.087	0.41
HCBW1-B	2640	2270	140	G14	0.86	369600	No	9.7	9.67	5	0.087	0.41
HCBW2	2640	2270	140	B14	0.86	369600	No	18.4	12.5	5	0.087	0.41
HCBW3	2640	2270	190	G19	0.86	501600	No	11.8	8.57	5	0.064	0.30
HCBW4	2640	2270	140	G14	0.86	369600	No	9.7	9.67	5	0.087	0.08
HCBW5	2640	2270	140	G14	0.86	369600	No	9.7	9.67	5	0	0.41
HCBW6	2640	2270	140	G14	0.86	550100	Yes	9.7	9.67	5	0.087	0.42
HCBW7	1020	2270	140	G14	2.23	142800	No	9.7	7.85	5	0.087	0.53

Note: Shadowed cells indicate differences with respect to the base-case (HCWB1).

**Table 2:** Mechanical properties of hollow concrete blocks.

Block	Length (mm)	Height (mm)	Width (mm)	Voids Ratio, $\nu_{r}$ (%)	Compressive Strength, $f_{\mathrm{cu}}$ (MPa)	Tensile Strength, $f_{\mathrm{tu}}$ (MPa)	Young Modulus, $E_{u}$ (MPa)
G14	390	190	140	45.2	9.7 {14.3%}	0.64 {15.9%}	6486 {24.5%}
G19	390	190	190	45.5	11.8 {23.3%}	0.61 {18.0%}	10,831 {73.9%}
B14	390	190	140	37.1	18.4 {5.48%}	1.89 {4.23%}	8274 {13.6%}

Note: Coefficients of variation in brackets.

**Table 3:** Results of stack-bond prism compression tests.

Block Type	HCB Compressive Strength, $f_{\mathrm{cu}}$ (MPa)	Block Width (mm)	Masonry	
			Compressive Strength, $f_{m}^{\prime }$ (MPa)	Young’s Modulus, $E_{m}$ (MPa)
G14	9.7 {14.3%}	140	9.67 {10.8%}	6078 {13.2%}
G19	11.8 {5.48%}	190	8.57 {3.9%}	7721 {29.9%}
B14	18.4 {23.3%}	140	12.47 {8.9%}	8174 {10.2%}

Note: Coefficients of variation in brackets.

**Table 4:** Characteristic results of tested BJR-PG-RM walls.

Wall	Load Direction	First Major Diagonal Crack				Maximum Lateral Load			
		Lateral Displacement, $\delta_{1\mathrm{cr}}$ (mm)	Drift, $\Delta_{1\mathrm{cr}}$ (%)	Lateral Force, $V_{1\mathrm{cr}}$ (kN)	Shear Stress, $\tau_{1\mathrm{cr}}$ ${}^{\left(a\right)}$ (MPa)	Lateral Displacement, $\delta_{V_{\max}}$ (mm)	Drift, $\Delta_{V_{\max}}$ (%)	Lateral Force, $V_{\max}$ (kN)	Shear Stress, $\tau_{g,\max}$ ${}^{\left(a\right)}$ (MPa)
HCBW1A	Push	1.90	0.08	180.1	0.49	12.44	0.55	338.5	0.90
	Pull	1.39	0.06	169.1	0.46	9.81	0.43	317.8	0.86
HCBW1B	Push	1.66	0.07	144.7	0.39	13.34	0.59	387.5	1.05
	Pull	3.33	0.15	215.2	0.58	13.64	0.60	381.8	1.03
HCBW2	Push	2.38	0.10	245.4	0.66	10.49	0.46	381	1.03
	Pull	1.25	0.06	226.3	0.61	8.46	0.37	346.2	0.94
HCBW3	Push	3.28	0.14	301.1	0.60	14.58	0.64	451.9	0.90
	Pull	3.63	0.16	253.2	0.50	14.23	0.63	421.1	0.84
HCBW4	Push	6.39	0.28	226.8	0.61	11.35	0.50	274.5	0.74
	Pull	5.47	0.24	213.6	0.58	11.65	0.51	243.4	0.66
HCBW5	Push	2.11	0.09	217	0.59	9.55	0.42	295.4	0.80
	Pull	2.77	0.12	228	0.62	7.48	0.33	279.9	0.76
HCBW6	Push	3.37	0.15	304.5	0.55	10.35	0.46	379.7	0.69
	Pull	3.23	0.14	282.6	0.51	9.76	0.43	386.1	0.70
HCBW7	Push	5.96	0.26	52.1	0.37	17.21	0.76	85.3	0.60
	Pull	6.58	0.29	54.5	0.38	19.49	0.86	82.8	0.58

${}^{\left(a\right)}$: Shear strength calculated based on the gross cross-sectional area.

**Table 5:** Parameters of idealized envelopes, ductility, and R factor.

Wall	Load Direction	$\delta_{y}$ (mm)	$\delta_{V_{\max}}$ (mm)	2$V_{\max}$ (kN)	$\mu_{d}$	2$\bar{\mu_{d}}$	R	$\bar{R}$
HCBW1-A	Push	5.47	12.44	338.5	2.27	2 2.21	1.88	21.85
	Pull	4.56	9.8	317.8	2.15		1.82	
HCBW1-B	Push	6.84	13.34	387.5	1.95	21.93	1.70	21.69
	Pull	7.18	13.64	381.8	1.90		1.67	
HCBW2	Push	4.64	10.49	381.0	2.26	22.66	1.88	22.07
	Pull	2.76	8.46	346.2	3.07		2.27	
HCBW3	Push	5.96	14.58	451.9	2.45	22.24	1.97	21.86
	Pull	6.98	14.23	421.1	2.04		1.75	
HCBW4	Push	6.16	11.35	274.5	1.84	22.3	1.64	21.88
	Pull	4.21	11.65	243.4	2.77		2.13	
HCBW5	Push	2.84	9.56	295.4	3.37	23.06	2.39	22.26
	Pull	2.72	7.48	279.9	2.75		2.12	
HCBW6	Push	3.46	10.35	379.7	2.99	22.57	2.23	22.02
	Pull	4.56	9.76	386.1	2.14		1.81	
HCBW7	Push	10.76	17.21	85.3	1.60	21.72	1.48	2 1.56
	Pull	10.58	19.49	82.8	1.84		1.64	

**Table 6:** Prediction of the resistance of the tested walls.

Wall	Experimental Resistance (kN)	Predicted Maximum Resistance (kN)				
		Shing et al. [3]	Psilla and Tassios [44]	CSA S304 [45]		TMS 402/602 [46]
				With Upper Limit	Without Upper Limit	
HCBW1A	338.46	459.04	309.00	251.92	256.54	282.18
HCBW1B	387.53	459.04	309.00	251.92	256.54	282.18
HCBW2	381.01	516.91	328.54	285.78	390.01	328.90
HCBW3	451.85	483.01	326.93	321.52	415.95	317.48
HCBW4	274.50	401.66	234.09	251.92	357.31	282.18
HCBW5	295.38	265.09	199.63	224.72	224.72	199.32
HCBW6	386.13	619.89	405.29	344.64	442.81	407.83
HCBW7	85.30	154.53	164.19	71.78	112.2	42.91

**Table 7:** Statistical parameters of prediction errors.

Statistical Parameter	$\left(V_{n}-V_{\exp}^{\max}\right)/V_{\exp}^{\max}$ (%)				
	Shing et al. [3]	Psilla and Tassios [44]	CSA S304 [45]		TMS 402/602 [46]
			With Upper Limit	Without Upper Limit	
Average	34.3	−2.4	−21.7	1.9	−20.1
Max	81.2	92.5	−8.2	31.6	5.6
Min	−10.3	−32.4	−35.2	−24.2	−49.7
SD	29.3	40.0	9.2	22.0	18.5
5th percentile	−4.3	−30.7	−32.9	−24.1	−43.7

## Abbreviations

The following abbreviations are used in this manuscript:

BJR	Bed-joint reinforcement
BBR	Bond-beam reinforcement
PG	Partial grouting
RM	Reinforced masonry

## Notation List

The following notation list describes the meaning of the symbols employed in Equations (6)–(18) and through the text:

$a_{i+1}$	Semi-amplitude of the displacement increment i+1
$A_{n}$	Net cross-section of the wall
$A_{sh}$	Area of a horizontal reinforcement (in this case two rods of ϕ4.2 mm)
$A_{sv}$	Area of a vertical reinforcement
$A_{svt},A_{sht}$	Total area of vertical and horizontal reinforcement, respectively
$A_{T}$	Total cross-section of the wall
$d^{\prime }$	Cover of reinforcement of the vertical edge
$d_{v}$	Effective depth of the wall
$d_{sv},d_{sh}$	Diameter of vertical and horizontal bars, respectively
$E_{m}$	Young’s modulus of masonry
$E_{env}$	Energy below the envelope curve up to its maximum resistance (in one selected load direction)
$E_{s,i}$	Dissipated energy in the cycle *i*
$E_{u}$	Young’s modulus of block units
$f_{cb}$	Compressive strength of brick
$f_{cg}$	Compressive strength of grout
$f_{cu}$	Compressive strength of the block units
$f_{m}^{\prime }$	Compressive strength of masonry
$f_{tm}$	Tensile strength of mortar
$f_{tg}$	Tensile strength of grout
$f_{tu}$	Tensile strength of block units
$f_{yv},f_{yh}$	Yielding strength of vertical and horizontal reinforcement, respectively
$f_{vk0}$	Shear resistance of masonry without axial load
$h_{w}$	Height of wall
$k_{bv},k_{bh}$	Term depending on the anchorage conditions of bars
$K_{sec,i}$	Lateral secant stiffness at the cycle *i*
$K_{sec,0}$	Initial lateral stiffness
$L_{w}$	Length of wall
*M*	Moment applied to the wall
*n*	Number of displacement increments between $\Delta_{0}$ and $\Delta_{m}$
*P*	Axial load applied to the wall
$s_{h}$	Vertical spacing of horizontal reinforcement
*t*	Gross width of the wall
$t_{n}$	Net width of the wall
*V*	Shear applied to the wall
$V_{1cr}$	Shear force at the formation of the first major diagonal crack in one load direction
$V_{max}$	Maximum shear force in one load direction
*R*	Seismic-force reduction factor
Δ	Inter-story drift
$\Delta_{0},\Delta_{m}$	Drift parameters of the displacement protocol
$\Delta_{1cr}$	Drift at detection of the first major diagonal crack in one load direction
$\Delta_{Vmax}$	Drift at maximum lateral force in one load direction
δ	Lateral displacement
$\delta_{1cr}$	Displacement at detection of the first major diagonal crack in one load direction
$\delta_{Vmax}$	Displacement at maximum lateral force in one load direction
$\delta_{b}$	Boundary condition factor (0.60 for cantilever support condition)
$\delta_{y}$	Deformation associated with the end of the idealized elastic behavior
$\delta_{u}$	Displacement of ultimate resistance
$\varepsilon_{y,HR}$	Yield strain of the horizontal reinforcement
$\mu_{d}$	Displacement ductility
γ	Type of masonry grouting factor (0.60 for PG-RM)
$\rho_{v},\rho_{h}$	Vertical and horizontal reinforcement ratio calculated over the cross-section area of the wall, respectively.
$\sigma_{n}$	Axial stress of wall calculated over the gross cross-section of the wall
$\nu_{r}$	Voids ratio of blocks
$\xi_{eq,i}$	Equivalent viscous damping ratio of the cycle *i*
$\tau_{1cr}$	Shear stress at the formation of the first major diagonal crack in one load direction
$\tau_{max}$	Maximum shear stress in one load direction
